# Ultrasonic Cavitation Transforms Organic Matter to Achieve Reduction of Excess Sludge and Recycling of Carbon Sources

**DOI:** 10.3390/toxics13110941

**Published:** 2025-10-31

**Authors:** Haohao Sun, Jie Li, Lu Zhuang, Yunian Zhang, Zhou Zhou, Jiayue Sun, Di Wang, Yanfang Ren, Xia Xu, Junyu He, Yingang Xue

**Affiliations:** 1 School of Environmental Science and Engineering, Changzhou University, Changzhou 213164, China; shh@cczu.edu.cn (H.S.);; 2 State Key Laboratory of Pollution Control and Resource Reuse, School of the Environment, Nanjing University, Nanjing 210093, China; 3 College of Urban Construction, Changzhou University, Changzhou 213164, China

**Keywords:** ultrasonic cavitation, sludge reduction, sludge resource utilization, organic matter conversion, denitrifying functional bacteria, pilot scale testing

## Abstract

Reducing the generation of excess sludge and achieving resource recovery are crucial for enhancing the economic efficiency and environmental sustainability of wastewater treatment plants (WWTPs). This study utilizes ultrasonic cavitation technology to transform organic matter into excess sludge to achieve sludge reduction and carbon source recovery. To this end, we systematically investigated the effects of various ultrasonic cavitation conditions on sludge reduction, organic matter conversion, and denitrification efficiency. The results showed that the optimal sludge reduction effect occurs at an original mixed liquid suspended solids (MLSS) of 10 g/L, under neutral and non-aerated conditions, reaching 15.07%. Ultrasonic cavitation treatment significantly enhanced the conversion efficiency of organic matter in the sludge, greatly increasing the concentration of organic matter in the supernatant, with soluble chemical oxygen demand (SCOD) maintained around 900 mg/L, thereby significantly improving the denitrification process. Furthermore, through magnetic-nanoparticle mediated isolation (MMI) and metagenomic sequencing analysis, the dominant denitrifying bacteria and their functional genes that utilize organic matter in the supernatant of ultrasonically treated sludge as a carbon source were identified. Finally, long-term pilot-scale operations further validated the practical application potential of ultrasonic cavitation technology for excess sludge reduction and resource utilization.

## 1. Introduction

With the acceleration of urbanization and industrialization, the number of wastewater treatment plants (WWTPs) has significantly increased [1]. This increase has led to a yearly rise in the production of excess sludge, making disposal issues more prominent [2]. Excess sludge has high moisture content (MC) [3], complex pathogen components, and contains a large number of toxic, harmful and perishable substances [4,5], which could be detrimental to the environment and human health without proper handling [6]. However, previous studies have indicated that excess sludge treatment and disposal account for approximately 50% of the operating costs of WWTPs [7,8]. It is a key factor affecting the economic benefits and sustainable development of WWTPs [9]. Therefore, there is an urgent need to reduce sludge production. In addition, Excess sludge contains large amounts of organic substances such as polysaccharide, protein, and lipid [10,11], and is known for its high carbon content (50~70%) [12,13], making it highly valuable in terms of resource recovery.

Common methods for sludge reduction include chemical (e.g., ozone oxidation, chemical conditioning), biological (e.g., anaerobic digestion), physical (e.g., concentration, dewatering, drying), and thermal (e.g., incineration, pyrolysis) [14,15,16]. In detail, chemical methods reduce sludge rapidly, but they are costly and prone to secondary pollution. Biological methods are renowned for their environmental friendliness and stability, but they require a longer treatment cycle. Physical methods are straightforward and effective; however, they have high energy consumption, limited reduction capacity, and require further treatment. Thermal treatment methods can effectively remove harmful substances, but their high cost and difficulty in controlling pollution limit their widespread application. Among these methods, anaerobic digestion (AD) is widely used [17,18], as it not only stabilizes and reduces sludge but also produces renewable energy sources such as biogas and hydrogen during fermentation [19,20], thereby achieving sludge resource utilization. Nevertheless, AD has drawbacks such as long processing time and high operational requirements [21]. It was reported that mesophilic anaerobic digestion typically needs a retention time of 15–30 days [22]. In some cases, the retention time can even extend to 65 days [23,24]. Consequently, methods for reducing and recycling sludge require improvement.

Ultrasonic cavitation technology has garnered considerable attention for its high efficiency, stability, and eco-friendliness in sludge and wastewater treatment [25,26]. This method effectively disrupts cell membranes, releasing cytoplasm rich in organic matter with potential for biological utilization [27,28]. As a primary component of sludge, organic matter can be transformed into carbon sources for organisms. Notably, Ref. [29] demonstrated that excess sludge thermal hydrolysate can serve as a premium carbon source for sulfate-reducing bacteria (SRB), presenting a novel solution for SO_4_^2−^ wastewater treatment and sludge resource utilization. Furthermore, ultrasonic cavitation can simplify complex organic matter into more biologically accessible compounds, thereby enhancing organic matter recycling [30,31,32]. Ultimately, this technology offers significant promise in sludge treatment, facilitating reduction, harmlessness, and recycling in line with sustainable development principles.

This study systematically investigated the effects of treating excess sludge under different ultrasonic cavitation conditions on sludge reduction, organic matter conversion, and the denitrification efficiency of supernatant as a carbon source. Unlike conventional studies that often focus solely on sludge disintegration, our work provides a comprehensive strategy that bridges sludge reduction with carbon source recovery for enhanced denitrification. Based on this, a pilot-scale long-term operation was conducted to evaluate its application potential. We also used magnetic-nanoparticle mediated isolation (MMI) technology to screen and enrich the sludge. Then, we analyzed the functional microbial community and functional gene in the sonicated sludge supernatant used as a carbon source for denitrification through metagenomic sequencing. Overall, this study demonstrates the feasibility of using ultrasonic cavitation technology to achieve reduction in excess sludge, recycle carbon sources, and proposes potential applications.

## 2. Material and Methods

### 2.1. Sludge Samples

The raw excess sludge was collected from the secondary sedimentation tank of a municipal wastewater treatment plant in Changzhou, China, which utilizes an A/A/O process and has a daily processing capacity of approximately 50,000 tons. The fundamental characteristics of the sludge are detailed in Table S1.

### 2.2. Experimental Procedures

#### 2.2.1. Batch Treatment of Excess Sludge via Ultrasonic Cavitation

The ultrasonic treatment equipment, detailed in Figure S1, operates at a frequency of 20 kHz with a rated output power of 600 W, and is equipped with a probe of 28 mm in diameter. In the experiment, 500 mL of sludge sample was placed into a 1000 mL beaker for ultrasonic treatment, with the probe positioned about 2 cm below the sludge surface. Experiments were conducted at room temperature with varying sludge concentrations (5, 10 and 15 g/L), ultrasonic durations (0, 4, 8, 12, 16 and 20 min), pH values (4, 7 and 10), and aeration conditions (normal and aerated). After the ultrasonic cavitation treatment, the sludge was collected, centrifuged, and the supernatant was stored for further analysis.

#### 2.2.2. Batch Experiment for Denitrification

The sludge subjected to ultrasonic cavitation under various conditions was centrifuged at 8000 rpm for 2 min. The resulting 200 mL supernatant was used as an external carbon source and transferred to a 500 mL conical flask. Then, 6 ± 0.2 g of raw excess sludge, previously centrifuged for 10 min, was added to the flask, along with 0.26 g of potassium nitrate. Nitrogen was bubbled through the mixture for 5 min to remove dissolved oxygen, and the flask was sealed with a rubber plug fitted with a sampling port. The flask was placed in a shaker set at 170 r/min and maintained at 25 ± 0.5 °C. Samples were taken every 4 h to measure nitrate nitrogen (NO_3_^−^-N) and total organic carbon (TOC) concentrations.

#### 2.2.3. Functional Bacteria Separated by MMI

The MMI approach [33] was employed to analyze the functional microbial community of the sonicated sludge supernatant as a carbon source. The synthesis of magnetic nanoparticles (MNPs) and the functionalization of biological sludge followed the previous descriptions [34,35,36], with some modifications. The specific operation is described in the Supporting Information (Text S1).

#### 2.2.4. Pilot-Scale Long-Term Operation

To assess the effectiveness of ultrasonic cavitation treatment in reducing and recycling sludge, a long-term pilot test was conducted using an “ultrasonic cavitation + upflow anaerobic sludge blanket (UASB)” system (Figure S2). The ultrasonic cavitation system comprised a 100 L tank and a 5 L ultrasonic reactor connected via pipelines to a centrifugal circulation pump, creating a recirculation treatment setup. The ultrasonic device was operated with hydraulic retention times (HRT) of 20, 40, 60, 80, 100 and 120 min. After the ultrasonic cavitation treatment, the sludge was transferred to a sedimentation tank and allowed to stand for 30 min. The supernatant then flowed into the UASB reactor, where it served as a carbon source for denitrification of synthetic wastewater with a NO_3_^−^-N concentration of 200 mg/L. The UASB reactor, with an effective volume of 20 L, had an HRT of 6 h, with water entering from the bottom and exiting from the top. The 180-day experiment involved monitoring various sludge and water quality indicators to evaluate the potential of ultrasonic cavitation technology and the operational stability of the pilot system.

### 2.3. Analytical and Data Methods

#### 2.3.1. Analytical Methods

Water samples were filtered through 0.45 µm filters, and the filtrate was stored at 4 °C for testing within one week [37]. The mixed liquid suspended solids (MLSS), mixed liquor volatile suspended solids (MLVSS) and MC were measured by weight method [38]. Other conventional parameters, including soluble chemical oxygen demand (SCOD), total nitrogen (TN), ammonia nitrogen (NH_3_^+^-N), and NO_3_^−^-N, were analyzed using standard methods [39]. TOC was analyzed using a TOC analyzer (Multi N/C 3100, Analytik Jena, Jena, Germany). All water quality tests were repeated three times to ensure experimental accuracy.

The improved thermal extraction method was used to extract tightly bound EPS (TB-EPS) from the sludge [40], detailed procedures are described in Text S2. The main components of EPS are proteins (PN) and polysaccharides (PS), which account for over 80% of the total extracellular polymeric substances (EPS) [41]. To simplify measurement, the sum of PN and PS content is used to represent the EPS content. PN was measured with the Coomassie Brilliant Blue method, and PS was tested using the anthrone-sulfuric acid method. Both were determined by ultraviolet spectrophotometer [42]. Additionally, we conducted three-dimensional fluorescence spectroscopy (3D-EEM) analysis on the sludge, scanning emission wavelengths (Em) from 280 to 550 nm and excitation wavelengths (Ex) from 220 to 400 nm [43].

#### 2.3.2. DNA Extraction

The DNA of each sludge sample was extracted using the FastDNA Spin Kit for soil (MP Biomedicals, Solon, OH, USA) following the manufacturer’s protocol. The purity and concentration of the extracted DNA were determined using a NanoDrop 2000 spectrophotometer (Thermo Fisher Scientific, Wilmington, DE, USA) and confirmed by agarose gel (1%) electrophoresis. The extracted DNA was stored at −20 °C.

#### 2.3.3. Metagenomic Sequencing

For metagenomic library construction, DNA samples were randomly sheared into fragments with an average size of about 350 bp. The resulting library was diluted to a concentration of 2 ng/μL, and its insert size was analyzed using an Agilent 2100 Bioanalyzer (Agilent Technologies, Santa Clara, CA, USA). Quantitative PCR (qPCR) was then performed to precisely determine the library concentration and ensure its quality. Sequencing was carried out on the Illumina HiSeq 4000 platform (Illumina, San Diego, CA, USA) using a paired-end 2 × 150 bp mode. The raw sequencing reads have been submitted to the NCBI Sequence Read Archive under accession number PRJNA1022869.

#### 2.3.4. Sequencing Data Analysis

Raw metagenomic reads were quality-filtered and adapter-trimmed using Trimmomatic. High-quality reads from each sample were assembled into contigs with MEGAHIT, after which BBMap was applied to align the reads back to the assemblies to calculate contig coverage. MetaBAT2 was subsequently used to reconstruct metagenome-assembled genomes (MAGs). The completeness and contamination of these MAGs were evaluated with CheckM based on lineage-specific marker genes. Taxonomic assignment was performed using GTDB-Tk according to the Genome Taxonomy Database, and reference genomes of the identified species were retrieved from NCBI. Functional gene prediction was conducted by Prodigal to identify open reading frames (ORFs), and the translated protein sequences were annotated against the AromaDeg database using BLASTP (Version 2.13.0+).

## 3. Results and Discussion

### 3.1. Effectiveness of Ultrasonic Cavitation Treatment in Reducing Excess Sludge

**Figure 1:**
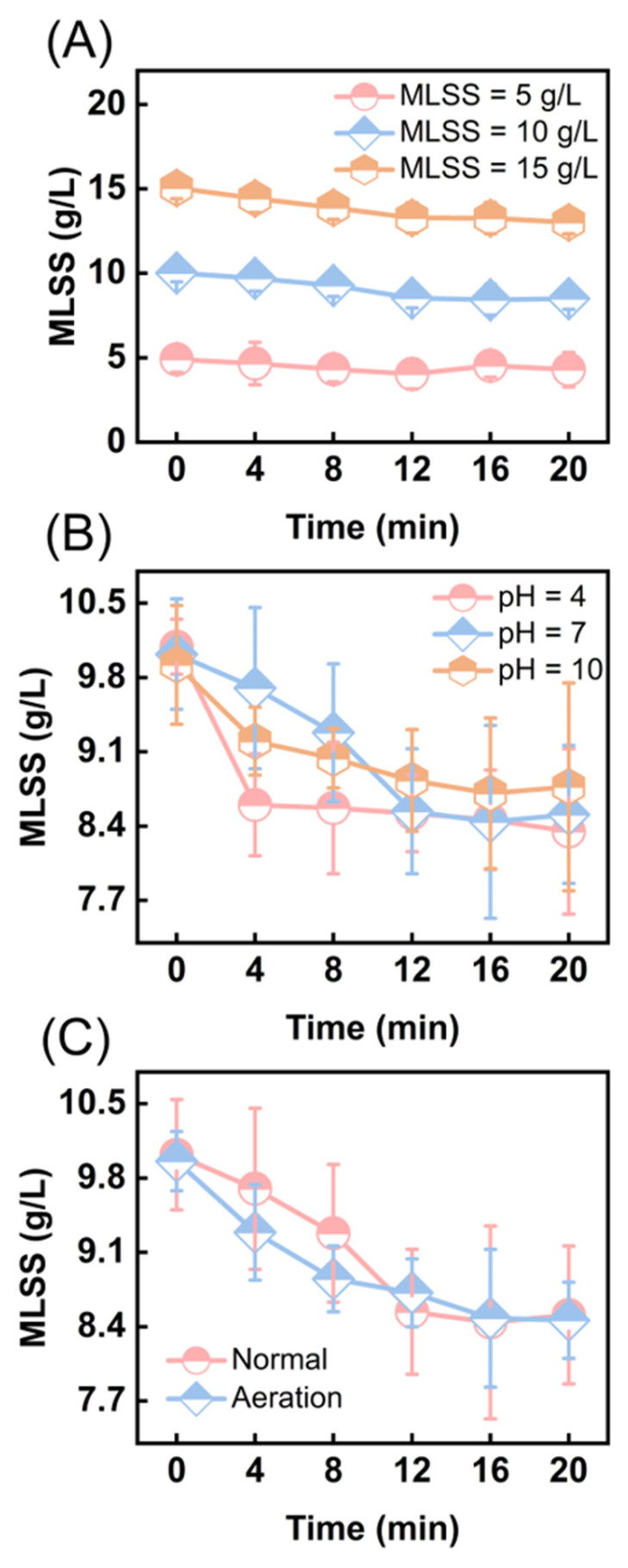
Effects of different conditions on MLSS changes during ultrasonic cavitation treatment of sludge. (**A**) Initial sludge concentrations; (**B**) pH values; (**C**) Aeration condition.

To investigate the effects of ultrasonic cavitation treatment on sludge reduction and its influencing factors, we conduct a quantitative analysis of sludge concentrations under varying conditions of sludge concentration, pH, and aeration, with different ultrasonic treatment durations. Overall, the sludge concentration decreases under various ultrasonic conditions, indicating that ultrasonic cavitation has an effective impact on sludge reduction (Figure 1). When the MLSS is 10 g/L, the reduction effect is most significant, reaching 15.07% (Figure 1A). This may be due to the obstruction of ultrasonic energy propagation at high sludge concentrations, which weakens the cavitation effect, leading to suboptimal sludge reduction [44]. Conversely, at low sludge concentrations, rapid collapse of cavitation bubbles and insufficient energy density result in dispersed cavitation effects, failing to effectively disrupt the sludge flocs and thus impacting the reduction efficiency [45]. Moreover, pH and aeration conditions have little impact on sludge reduction. The sludge reduction effect is more pronounced during the initial phase across the three different pH levels but converges after 12 min (Figure 1B). At pH 4 and pH 10, the sludge reduction trends showed similar patterns. During the first 4 min, the MLSS in both groups decreased rapidly. Then, the reduction rate gradually slowed, but the overall reduction effect at pH 4 was stronger than that at pH 10. This was mainly because under acidic conditions, OH radicals have stronger oxidation ability, allowing them to act more effectively on organic matter and microbial cell walls in the sludge. This process breaks cell structures and promotes sludge decomposition and reduction [46]. In addition, as the ultrasonic treatment time increases, the concentration change trends under the two aeration conditions are consistent. The sludge reduction under aeration conditions is 15.06%, which is not significantly different from conventional conditions (Figure 1C). Therefore, considering the reduction effect and economic cost comprehensively, it is more reasonable to select an initial MLSS of 10 g/L, a neutral pH value, and no aeration conditions for ultrasonic cavitation treatment of excess sludge.

We previously examined the impact of ultrasonic cavitation on sludge reduction. Dewatering is essential in sludge treatment, as it can significantly reduce sludge volume [47,48]. However, the effects of ultrasound on sludge dewatering remain ambiguous and warrant further exploration. Figure S3 illustrates the changes in sludge moisture content (MC) under various conditions, showing an initial decrease followed by an increase. Overall, sludge MC decreased across different ultrasonic settings, potentially due to ultrasonic cavitation disrupting flocs and cellular structures, thereby releasing water and facilitating the aggregation of smaller sludge particles [49], which enhances dewatering efficiency. Nevertheless, as the treatment time extends, sludge decomposes excessively, releasing plenty of organics into the supernatant. This increases viscosity, thereby decreasing dewatering performance of the sludge [50]. Furthermore, while factors like pH and aeration have minimal influence on sludge MC, sludge concentration plays a more critical role. Specifically, at varying sludge concentrations, the lowest MC was achieved after 12 min of ultrasonic cavitation treatment, with values of 73.2%, 68.5% and 71.9%, respectively. These findings suggest that ultrasonic cavitation technology can effectively reduce sludge, but the ultrasonic treatment time and MLSS must be controlled within a certain range to maintain good dewatering performance.

### 3.2. Conversion of Organic Matter in Excess Sludge Through Ultrasonic Cavitation Treatment

**Figure 2:**
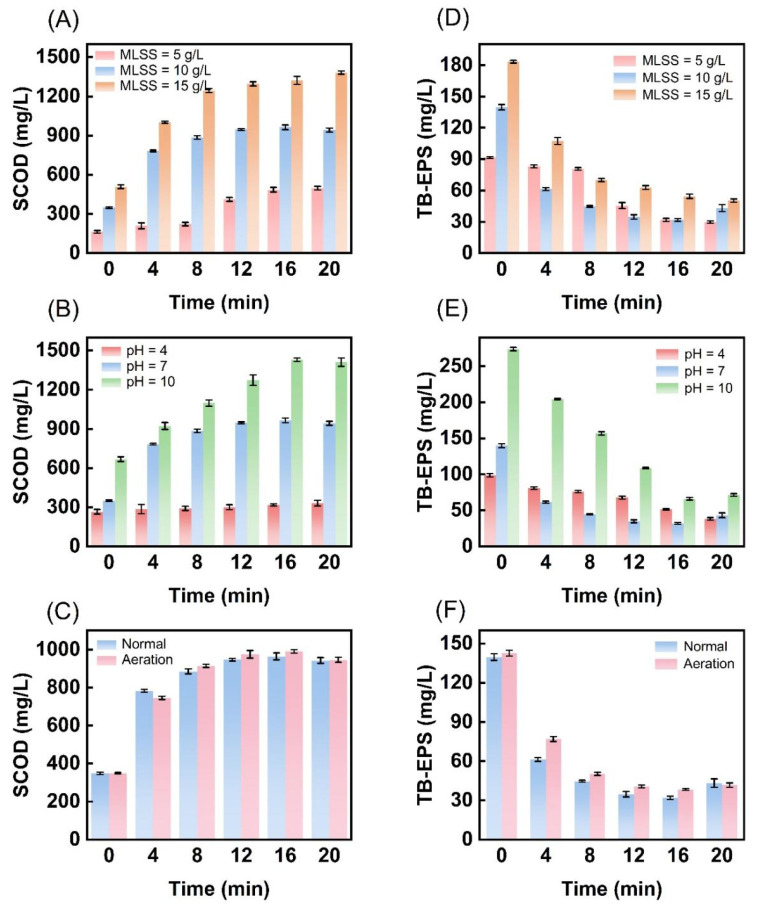
Effects of different ultrasonic cavitation conditions on the transformation of organic matter into excess sludge. (**A**) Changes in SCOD under different initial sludge concentrations, (**B**) Changes in SCOD under different pH values, (**C**) Changes in SCOD under different aeration conditions, (**D**) Changes in TB-EPS under different initial sludge concentrations, (**E**) Changes in TB-EPS under different pH values, (**F**) Changes in TB-EPS under different aeration conditions.

The observed reduction in sludge may result from the conversion of organic matter, prompting our investigation into the SCOD in the sludge supernatant. As shown in Figure 2, SCOD concentration rises with extended ultrasonic cavitation treatment time but eventually plateaus. Notably, varying initial concentrations and pH significantly influence SCOD levels. As sludge concentration and pH increase, SCOD can approach a maximum of nearly 1500 mg/L (Figure 2A–C). Initially, cavitation generates strong shear forces and high temperatures [51], disrupting floc structures and extracellular polymeric substances (EPS), which leads to a rapid increase in SCOD. EPS can be classified into soluble EPS (S-EPS), loosely bound EPS (LB-EPS), and tightly bound EPS (TB-EPS) based on their spatial distribution and compactness [52]. Ultrasonic treatment disrupts sludge floc structures, releasing TB-EPS into the liquid phase, while a decline in TB-EPS concentration indicates organic matter conversion. After 12 min, SCOD concentration stabilizes (Figure 2A–C), likely due to the protective multi-layer peptidoglycan walls of microbial cells, which inhibit lysis (Figure 2D–F) [53] and the release of the most readily accessible organic matter.

**Figure 3:**
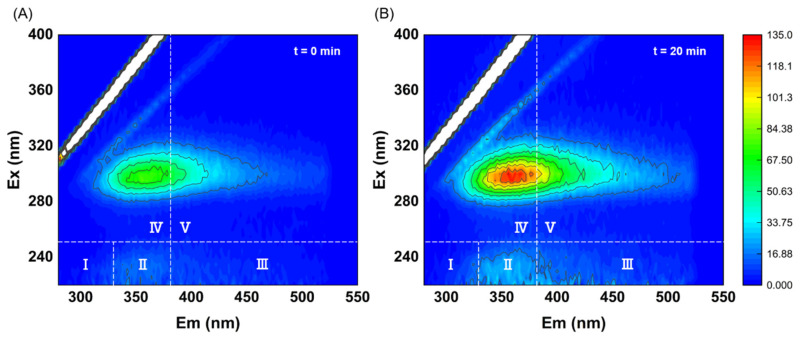
Characteristics of organic matter components in sludge supernatant. (**A**) Raw sludge; (**B**) Sludge treated with ultrasound cavitation for 20 min.

To further investigate the effect of ultrasonic cavitation treatment on the conversion of organic matter in sludge, 3D-EEM tests were conducted on two groups of sludge before and after ultrasonic treatment. At the same dilution ratio, the fluorescence intensity reflects the relative content of certain organic substances in the system; the stronger the fluorescence intensity, the higher the proportion of that substance in the system [54]. According to previous studies, the fluorescence spectrum can be divided into five regions, as shown in Table S2. Among them, regions I, II and IV represent microbially degradable substances, whereas regions III and V are substances that are not utilizable by microorganisms. As shown in Figure 3A, fluorescence peaks are present in regions II, IV and V of the raw sludge supernatant, with the peak in region IV being the most prominent. This depicts that the raw sludge supernatant is mainly composed of soluble microbial by-product, with relatively few tryptophan-like protein and humic acid-like protein. After 20 min of ultrasonic cavitation treatment, the three-dimensional fluorescence spectrum characteristics of fluorescent organic matter in the sludge supernatant changed significantly. The fluorescence of soluble microbial by-product, tryptophan-like protein, and humic acid-like protein increased markedly (Figure 3B). Additionally, new fluorescence peaks corresponding to fulvic acid-like organics were generated. Such results suggest that ultrasonic cavitation treatment effectively breaks down sludge and generates easily biodegradable dissolved organic matter. This process significantly enhanced the biodegradability of carbon sources in the supernatant, making it suitable for use as a denitrification carbon source.

### 3.3. Organic Matter in the Sludge Supernatant After Ultrasonic Cavitation as a Carbon Source for Denitrification

**Figure 4:**
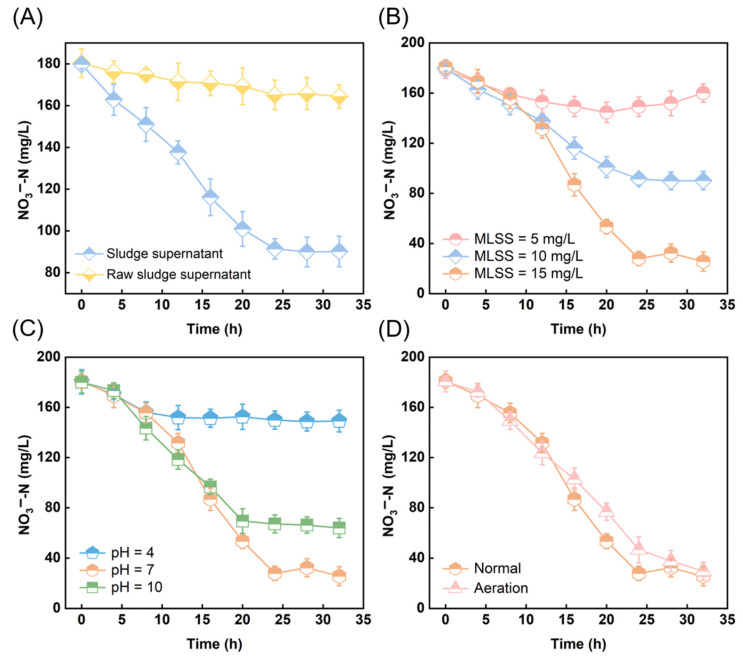
Denitrification effects under different ultrasonic cavitation conditions. (**A**) Ultrasonic cavitation and non-ultrasonic cavitation for sludge treatment; (**B**) Initial sludge concentrations; (**C**) pH values; (**D**) Aeration condition.

Based on the analysis, the optimal parameters for sludge reduction are MLSS = 10 g/L, pH = 7, and there is no aeration. Before proceeding with the denitrification batch experiment, we need to assess the feasibility of using the supernatant from this sludge as a carbon source for denitrification, comparing it to raw sludge supernatant. As shown in Figure 4A, nitrate is gradually degraded over time under both carbon source conditions, with TOC concentration trends mirroring the NO_3_^−^-N degradation trends (Figure S4A). Notably, the NO_3_^−^-N concentration is lowest when using the supernatant from sludge treated with ultrasound for 12 min. The final NO_3_^−^-N removal rates for these carbon sources are 8.87% and 49.83%, respectively. These results indicate that both carbon sources are viable for denitrification, with the ultrasonically treated sludge supernatant significantly enhancing the process.

For the purpose of optimizing operating parameters and achieving the best denitrification effect, we systematically studied the impact of ultrasonic cavitation sludge on denitrification under different conditions (initial sludge concentrations, pH values, and aeration conditions). Figure 4B depicts that NO_3_^−^-N gradually degrades over time under different initial sludge concentration conditions. As the sludge concentration increases, the degradation rate of NO_3_^−^-N accelerates, with the fastest degradation observed at MLSS = 15 g/L, where NO_3_^−^-N is nearly completely removed within 24 h. Higher sludge concentrations provide abundant microbial resources and favorable reaction conditions, enhancing the denitrification rate and carbon source utilization, thereby significantly improving the removal efficiency of nitrate nitrogen, which is consistent with the TOC trend (Figure S4B). Additionally, the denitrification removal efficiency varies significantly across different sludge concentrations, with the nitrogen removal rates of the three experiments being 10.39%, 49.83% and 85.85%, respectively. From Figure S4C, it can be seen that pH affects the ultrasound process, thereby influencing the utilization of carbon sources in the supernatant. Although the pH of all samples was adjusted to neutral before the denitrification experiment, the analysis results showed that under acidic conditions, the TOC concentration in the supernatant was low, being only 159.2 mg/L at the initial time, and the carbon source utilization was also poor, with a final denitrification rate of only 17.33% (Figure 4C). This could be attributed to the inhibition of carbon source release and utilization by ultrasound treatment under acidic conditions. In addition, the TOC content in the supernatant under alkaline conditions was higher than that under neutral conditions. Yet the denitrification rate under neutral conditions (85.85%) was higher than that under alkaline conditions (64.44%). This phenomenon may be due to the fact that under strong alkaline conditions, ultrasound treatment damaged the carbon sources in the supernatant, reducing their availability and thereby affecting the denitrification efficiency. Furthermore, the NO_3_^−^-N removal rate and TOC utilization rate in the aeration system do not differ significantly from those under normal conditions (Figure 4D and Figure S4D). In summary, MLSS = 15 g/L, neutral and non-aeration conditions are more conducive to the denitrification process and also contribute to energy efficiency and cost reduction.

### 3.4. Functional Bacteria and Genes Utilizing Organic Matter in Sludge Supernatant After Ultrasonic Cavitation Treatment

To gain a deeper understanding of the impact of ultrasonic cavitation treatment on the sludge microbial community structure in denitrification experiments utilizing the supernatant as a carbon source, we conducted an MMI experiment using three ultrasonic sludge supernatants (with ultrasound durations of 4, 8 and 12 min) as carbon sources to isolate functional bacteria. Subsequently, we explored the composition of these functional bacteria and their associated functional genes through metagenomic sequencing.

**Figure 5:**
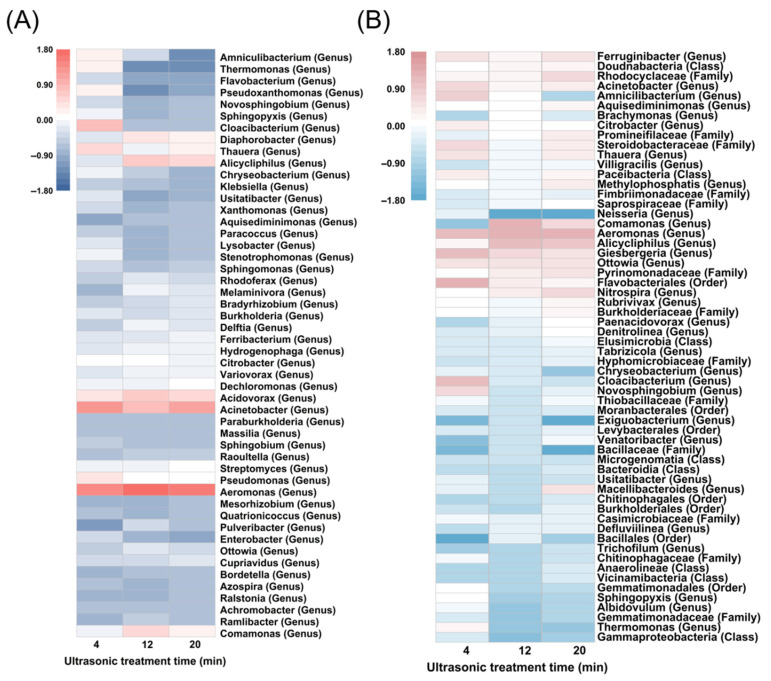
Effects of carbon sources obtained from excess sludge treated with different ultrasonic cavitation times on microbial community structure. (**A**) Changes in microbial composition at the genus level (top 50 dominant bacterial genera); (**B**) Changes in bacteria carrying denitrification genes.

The results in Figure 5 illustrate the impact of ultrasonic treatment duration on the sludge microbial community structure and the presence of bacteria carrying denitrification function genes. Figure 5A presents the variations in bacterial community structure under different sonication times (4, 12 and 20 min). Obviously, *Acidovorax*, *Acinetobacter*, and *Aeromonas* are the main dominant genera in the three samples. Previous studies have shown that these three genera play an important role in denitrification [55,56,57]. Among them, *Acidovorax* is abundant in activated sludge, which is not only capable of denitrification using a variety of carbon and nitrogen sources, but also has a certain capacity of organic matter degradation [58] (Huang et al., 2023). Importantly, as the duration of ultrasonic cavitation treatment increased, the relative abundance of genera with denitrification and nitrogen removal capabilities exhibited a marked increase, including *Alicycliphilus*, *Diaphorobacter*, and *Comamonas* [59,60,61,62]. However, there are also species with significantly reduced abundance, such as *Aeromonas* and *Pseudomonas*. This variation may indicate that sonication time affects the carbon source composition, with some bacterial genera becoming more dominant in sonication-generated carbon source conditions, leading to their increased abundance.

Figure 5B examines the impact of carbon sources generated from the ultrasonic treatment of sludge on the bacterial community structure associated with denitrification functional genes. The results indicate that the overall abundance of bacteria carrying denitrification functional genes across the three samples showed little variation, with all groups containing a significant number of bacteria capable of organic matter degradation (Tables S3–S5). Notably, the abundance of certain denitrifying bacteria exhibited varying degrees of change depending on treatment duration. Longer treatment times correlated with an increase in the abundance of specific bacteria carrying denitrification functional genes, likely due to the enhanced carbon sources provided by ultrasonic treatment. For instance, the abundance of *Ferruginibacter* increased at 20 min, suggesting that this genus has greater denitrification potential under carbon-rich conditions post-treatment. Figure 5B highlights the selective effects of sonication on bacteria with denitrification functional genes, revealing that extended sonication promotes the growth and activity of these bacteria by providing a supportive carbon source for the denitrification process. Additionally, the abundance of certain bacterial genera, such as *Cloacibacterium*, decreased, possibly due to inhibition by the carbon sources [63].

As ultrasonic treatment time increases, the soluble organic matter and carbon sources generated in the sludge may become more abundant and diverse [64], influencing the adaptability and competitiveness of various microbial communities [65,66]. The carbon sources produced not only significantly impact the overall bacterial community structure but also exert a selective effect on bacteria that carry denitrification functional genes [67]. By effectively controlling the ultrasonic treatment duration, it is possible to regulate the microbial community in the sludge and optimize the proliferation of denitrifying bacteria, thereby enhancing denitrification efficiency in sewage treatment systems. This finding holds significant implications for optimizing sewage treatment processes.

### 3.5. Long-Term Pilot-Scale Treatment of Excess Sludge by Ultrasonic Cavitation

**Figure 6:**
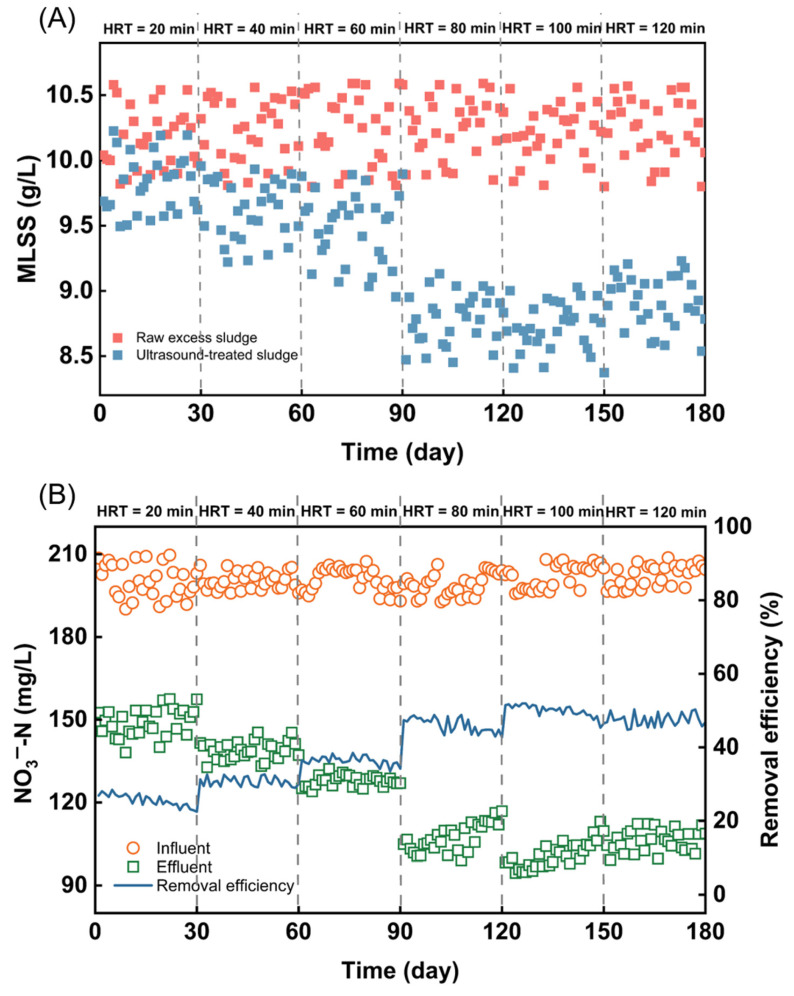
The reduction and recycling effects of excess sludge by pilot-scale ultrasonic cavitation treatment. (**A**) MLSS; (**B**) Concentration and removal efficiency of NO_3_^–^-N.

To evaluate the application potential of ultrasonic cavitation technology, we conducted a long-term pilot-scale experiment. The system processes 100 L of sludge per batch, with raw MLSS concentrations ranging from 9.8 to 10.6 g/L and MLVSS from 5.6 to 6.5 g/L. Figure 6 illustrates the effects of pilot-scale ultrasonic cavitation on sludge reduction and recovery. As the hydraulic retention time (HRT) in the reactor increased, both MLSS and MLVSS gradually decreased (Figure 6A and Figure S5), due to the conversion of organic matter. Ultrasonic cavitation released solid organic matter into the liquid phase, resulting in an increase in soluble chemical oxygen demand (SCOD) in the supernatant (Figure S6). Notably, at HRTs of 80 and 100 min, the sludge reduction effect was significant, maintaining around 14%, while further increases in HRT did not enhance sludge reduction. Additionally, to investigate the denitrification and nitrogen removal capacity of the supernatant from ultrasound-treated sludge, a long-term denitrification experiment was performed using a UASB reactor with the supernatant as the carbon source. Figure 6B shows the changes in NO_3_^−^-N concentration and its removal effects in both influent and effluent. As the HRT increased, the NO_3_^−^-N concentration in the effluent significantly decreased. This reduction may be attributed to the extended ultrasonic treatment time, which enhances the conversion of organic components in the sludge into SCOD [68]. When the HRT reached 100 min, the NO_3_^−^-N removal rate stabilized at around 50%. This stabilization likely occurs because the amount of organic matter in the sludge that can be converted by ultrasound is limited [69]. Overall, the long-term pilot experiment demonstrated the stability and ultimate application potential of ultrasonic cavitation technology for sludge reduction and resource recovery.

## 4. Conclusions

In conclusion, this study demonstrates the effective application of ultrasonic cavitation technology for reducing excess sludge generation and recovering valuable resources in wastewater treatment plants (WWTPs). The research identified optimal conditions for achieving a sludge reduction of 15.07% at an initial mixed liquid suspended solids (MLSS) concentration of 10 g/L, under neutral and non-aerated conditions. Ultrasonic treatment markedly improved the conversion of organic matter, leading to an increase in soluble chemical oxygen demand (SCOD) around 900 mg/L, which in turn enhanced denitrification efficiency. Additionally, the identification of key denitrifying bacteria and their functional genes utilizing the organic matter from treated sludge provides insights into microbial dynamics in the process. Long-term pilot-scale operations further confirmed the technology’s stability and practical viability for excess sludge management, positioning ultrasonic cavitation as a promising solution for enhancing both the economic and environmental sustainability of WWTPs.

## Data Availability

The original contributions presented in this study are included in the article. Further inquiries can be directed to the corresponding author.

## Supplementary Materials

The following supporting information can be downloaded at https://www.mdpi.com/article/10.3390/toxics13110941/s1: Text S1. The synthesis of MNPs and the functionalization of biological sludge; Text S2. Extraction of TB-EPS; Figure S1. Schematic diagram of the ultrasonic cavitation experimental device: (1) Ultrasonic generator; (2) Reaction tank; (3) Ultrasonic transducer; (4) Ultrasonic probe; Figure S2. Schematic diagram of pilot-scale ultrasonic cavitation experiment process; Figure S3. Changes in MC under different conditions; Figure S4. Changes in TOC concentration. (A) Feasibility of using sludge supernatant as a carbon source; (B) Initial sludge concentrations; (C) pH values; (D) Aeration condition; Figure S5. The effect of pilot-scale ultrasonic cavitation treatment on MLVSS; Figure S6. The effect of pilot-scale ultrasonic cavitation treatment on SCOD; Table S1. The basic characteristics of the raw excess sludge; Table S2. Excitation and emitting (Ex/Em) wavelengths of fluorescence region; Table S3. Host bacteria carrying organic degradation functional genes in sample 1; Table S4. Host bacteria carrying organic degradation functional genes in sample 2; Table S5. Host bacteria carrying organic degradation functional genes in sample 3.

## References

[1] Zhang Q.H., Yang W.N., Ngo H.H., Guo W.S., Jin P.K., Dzakpasu M., Yang S.J., Wang Q., Wang X.C., Ao D. (2016). Current Status of Urban Wastewater Treatment Plants in China. Environ. Int..

[2] Chang H., Zhao Y., Li X., Damgaard A., Christensen T.H. (2022). Review of Inventory Data for the Biological Treatment of Sewage Sludge. Waste Manag..

[3] Huang J., Liang J., Yang X., Zhou J., Liao X., Li S., Zheng L., Sun S. (2020). Ultrasonic Coupled Bioleaching Pretreatment for Enhancing Sewage Sludge Dewatering: Simultaneously Mitigating Antibiotic Resistant Genes and Changing Microbial Communities. Ecotoxicol. Environ. Saf..

[4] Cheng Y., Tian K., Xie P., Ren X., Li Y., Kou Y., Chon K., Hwang M.-H., Ko M.-H. (2022). Insights into the Minimization of Excess Sludge Production in Micro-Aerobic Reactors Coupled with a Membrane Bioreactor: Characteristics of Extracellular Polymeric Substances. Chemosphere.

[5] Zhang R., Mao Y., Meng L. (2021). Excess Sludge Cell Lysis by Ultrasound Combined with Ozone. Sep. Purif. Technol..

[6] Hoang S.A., Bolan N., Madhubashani A.M.P., Vithanage M., Perera V., Wijesekara H., Wang H., Srivastava P., Kirkham M.B., Mickan B.S. (2022). Treatment Processes to Eliminate Potential Environmental Hazards and Restore Agronomic Value of Sewage Sludg: A Review. Environ. Pollut..

[7] Fida Z., Price W.E., Pramanik B.K., Dhar B.R., Kumar M., Jiang G., Hai F.I. (2021). Reduction of Excess Sludge Production by Membrane Bioreactor Coupled with Anoxic Side-Stream Reactors. J. Environ. Manag..

[8] Lambert N., Van Aken P., Smets I., Appels L., Dewil R. (2022). Performance Assessment of Ultrasonic Sludge Disintegration in Activated Sludge Wastewater Treatment Plants under Nutrient-Deficient Conditions. Chem. Eng. J..

[9] Cheng C., Zhou Z., Niu T., An Y., Shen X., Pan W., Chen Z., Liu J. (2017). Effects of Side-Stream Ratio on Sludge Reduction and Microbial Structures of Anaerobic Side-Stream Reactor Coupled Membrane Bioreactors. Bioresour. Technol..

[10] Li X., Jin W., Du Y., Tan Z., Liu R., Xiao R., Wang J., Zhou L. (2021). Electron Beam Irradiation Effect on the Physicochemical Characteristics of Municipal Excess Sludge. Radiat. Phys. Chem..

[11] Xie J., Xin X., Ai X., Hong J., Wen Z., Li W., Lv S. (2022). Synergic Role of Ferrate and Nitrite for Triggering Waste Activated Sludge Solubilisation and Acidogenic Fermentation: Effectiveness Evaluation and Mechanism Elucidation. Water Res..

[12] Du R., Li C., Liu Q., Fan J., Peng Y. (2022). A Review of Enhanced Municipal Wastewater Treatment through Energy Savings and Carbon Recovery to Reduce Discharge and CO_2_ Footprint. Bioresour. Technol..

[13] Banu J.R., Kumar G., Tyagi V.K., Bajhaiya A.K., Gugulothu P., Gunasekaran M. (2022). Biohydrogen Production from Waste Activated Sludge through Thermochemical Mechanical Pretreatment. Bioresour. Technol..

[14] Bosco Mofatto P.M., Cosenza A., Di Trapani D., Mannina G. (2024). Investigation of Intermittent Aeration and Oxic Settling Anaerobic Process Combination for Nitrogen Removal and Sewage Sludge Reduction. Chemosphere.

[15] Mannina G., Barbara L., Cosenza A., Wang Z. (2023). Treatment and disposal of sewage sludge from wastewater in a circular economy perspective. Current Developments in Biotechnology and Bioengineering.

[16] Zhao Y., Yang Z., Niu J., Du Z., Federica C., Zhu Z., Yang K., Li Y., Zhao B., Pedersen T.H. (2023). Systematic Analysis of Sludge Treatment and Disposal Technologies for Carbon Footprint Reduction. J. Environ. Sci..

[17] Cao X., Li S., Liu C. (2025). Effect of Lysozyme Combined with Hydrothermal Pretreatment on Excess Sludge and Anaerobic Digestion. J. Environ. Sci..

[18] Nayeri D., Mohammadi P., Bashardoust P., Eshtiaghi N. (2024). A Comprehensive Review on the Recent Development of Anaerobic Sludge Digestions: Performance, Mechanism, Operational Factors, and Future Challenges. Results Eng..

[19] Qin S., Zhang D., Wang J., Liang M., Chen W., Zhang T., Lu X., Li L., Wu X., Zan F. (2024). In-Situ Sulfite Treatment Promotes Solid Reduction during Aerobic Digestion of Waste Activated Sludge: Feasibility for Small-scale Wastewater Treatment Plants. Bioresour. Technol..

[20] Zou X., He J., Zhang P., Pan X., Zhong Y., Zhang J., Wu X., Li B., Tang X., Xiao X. (2022). Insights into Carbon Recovery from Excess Sludge through Enzyme-catalyzing Hydrolysis Strategy: Environmental benefits and carbon-emission reduction. Bioresour. Technol..

[21] Wang Y., Zheng K., Ding J., Guo H., Chen X., Zhu T., Sun P., Liu Y. (2023). Ultrasonic Radiation Enhances Percarbonate Oxidation for Improving Anaerobic Digestion of Waste Activated Sludge. Chem. Eng. J..

[22] Mao C., Feng Y., Wang X., Ren G. (2015). Review on Research Achievements of Biogas from Anaerobic Digestion. Renew. Sust. Energy Rev..

[23] Rawoof S.A.A., Kumar P.S., Vo D.-V.N., Subramanian S.J.E.C.L. (2020). Sequential Production of Hydrogen and Methane by Anaerobic Digestion of Organic Wastes: A Review. Environ. Chem. Lett..

[24] Saha M., Eskicioglu C., Marin J. (2011). Microwave, Ultrasonic and Chemo-Mechanical Pretreatments for Enhancing Methane Potential of Pulp Mill Wastewater Treatment Sludge. Bioresour. Technol..

[25] Li W., Yu N., Fang A., Liu B., Ren N., Xing D. (2019). Co-Treatment of Potassium Ferrate and Ultrasonication Enhances Degradability and Dewaterability of Waste Activated Sludge. Chem. Eng. J..

[26] Wolny L., Wolski P. (2021). Ultrasounds Energy as an Agent of Polyelectrolyte Modification Prior to Sewage Sludge Conditioning. Energies.

[27] Akin B. (2008). Waste Activated Sludge Disintegration in an Ultrasonic Batch Reactor. Clean-Soil Air Water.

[28] Lippert T., Bandelin J., Schlederer F., Drewes J.E., Koch K. (2019). Impact of Ultrasound-Induced Cavitation on the Fluid Dynamics of Water and Sewage Sludge in Ultrasonic Flatbed Reactors. Ultrason. Sonochem..

[29] Cui T.T., Zhou J.T., Jin R.F., Li X. (2024). Treatment of Sulfate Wastewater by Sulfate-Reducing Bacteria with Residual Sludge Thermal Alkaline Hydrolysate as Carbon Source. Environ. Eng..

[30] Caricasole P., Provenzano M.R., Hatcher P.G., Senesi N. (2010). Chemical Characteristics of Dissolved Organic Matter during Composting of Different Organic Wastes Assessed by 13C CPMAS NMR Spectroscopy. Bioresour. Technol..

[31] Mehrdadi N., Kootenaei F.G. (2018). An Investigation on Effect of Ultrasound Waves on Sludge Treatment. Energy Procedia.

[32] Zhang L., Zhang M., Kang G., Xu Z. (2025). Ultrasound Activated Peroxymonosulfate Oxidation for Advanced Water Treatment: Cavitation Mechanism, Free Radical Generation and Process Simulation. Ultrason. Sonochem..

[33] Zhang D.Y., Berry J.P., Zhu D., Wang Y., Chen Y., Jiang B., Huang S., Langford H., Li G.H., Davison P.A. (2015). Magnetic Nanoparticle-Mediated Isolation of Functional Bacteria in a Complex Microbial Community. ISME J..

[34] Li J., Luo C., Zhang G., Zhang D. (2018). Coupling Magnetic-Nanoparticle Mediated Isolation (MMI) and Stable Isotope Probing (SIP) for Identifying and Isolating the Active Microbes Involved in Phenanthrene Degradation in Wastewater with Higher Resolution and Accuracy. Water Res..

[35] Lin Z., Xu Y., Zhen Z., Fu Y., Liu Y., Li W., Luo C., Ding A., Zhang D. (2015). Application and Reactivation of Magnetic Nanoparticles in Microcystis aeruginosa Harvesting. Bioresour. Technol..

[36] Zhang D.Y., Fakhrullin R.F., Özmen M., Wang H., Wang J., Paunov V.N., Li G.H., Huang W.E. (2010). Functionalization of Whole-Cell Bacterial Reporters with Magnetic Nanoparticles. Microb. Biotechnol..

[37] You B.-C., Huang C.-C., Chuang S.-H. (2023). The Characteristics of Stepwise Ultrasonic Hydrolysates of Sludge for Enhancing Denitrification. Bioresour. Technol..

[38] Zhang Y., Ling Z., Zhao M., Sha L., Li C., Lu X. (2023). Investigation of the Properties and Mechanism of Activated Sludge in Acid-Magnetic Powder Conditioning and Vertical Pressurized Electro-Dewatering (AMPED) Process. Sep. Purif. Technol..

[39] Rice E.W., Bridgewater L., American Public Health Association (2012). Standard Methods for the Examination of Water and Wastewater.

[40] Zhang H., Xu Y., Bin L., Li P., Fu F., Huang S., Tang B. (2023). Rapid Granulation of Aerobic Granular Sludge in an Integrated Moving Bed Biofilm Reactor-membrane Bioreactor: Effects and Mechanism of *Streptomyces cellulosus* Microspheres. Bioresour. Technol. Rep..

[41] Flemming H.-C., Wingender J. (2001). Relevance of Microbial Extracellular Polymeric Substances (EPSs)—Part I: Structural and Ecological Aspects. Water Sci. Technol..

[42] Li Y., Pan L., Zhu Y., Yu Y., Wang D., Yang G., Yuan X., Liu X., Li H., Zhang J. (2019). How does zero valent iron activating peroxydisulfate improve the dewatering of anaerobically digested sludge?. Water Res..

[43] Li L., Wang Y., Zhang W., Yu S., Wang X., Gao N. (2020). New Advances in Fluorescence Excitation–Emission Matrix Spectroscopy for the Characterization of Dissolved Organic Matter in Drinking Water Treatment: A Review. Chem. Eng. J..

[44] Parandoush S., Mokhtarani N. (2022). Reducing Excess Sludge Volume in Sequencing Batch Reactor by Integrating Ultrasonic Waves and Ozonation. J. Environ. Manag..

[45] Gao J., Liu Y., Yan Y., Wan J., Liu F. (2021). Promotion of Sludge Process Reduction Using Low-Intensity Ultrasonic Treatment. J. Clean. Prod..

[46] Sun X., Xu K., Chatzitakis A., Norby T. (2021). Photocatalytic Generation of Gas Phase Reactive Oxygen Species from Adsorbed Water: Remote Action and Electrochemical Detection. J. Environ. Chem. Eng..

[47] Cao B., Zhang T., Zhang W., Wang D. (2021). Enhanced Technology Based for Sewage Sludge Deep Dewatering: A Critical Review. Water Res..

[48] Wu B.R., Dai X.H., Chai X.L. (2020). Critical Review on Dewatering of Sewage Sludge: Influential Mechanism, Conditioning Technologies and Implications to Sludge Re-Utilizations. Water Res..

[49] Qi Y., Chen J., Xu H., Wu S., Yang Z., Zhou A., Hao Y. (2024). Optimizing Sludge Dewatering Efficiency with Ultrasonic Treatment: Insights into Parameters, Effects, and Microstructural Changes. Ultrason. Sonochemistry.

[50] Zhang P., Zhang G., Wang W. (2007). Ultrasonic Treatment of Biological Sludge: Floc Disintegration, Cell Lysis and Inactivation. Bioresour. Technol..

[51] Huan L., Yiying J., Mahar R.B., Zhiyu W., Yongfeng N. (2009). Effects of Ultrasonic Disintegration on Sludge Microbial Activity and Dewaterability. J. Hazard. Mater..

[52] Shi Y., Huang J., Zeng G., Gu Y., Chen Y., Hu Y., Tang B., Zhou J., Yang Y., Shi L. (2017). Exploiting Extracellular Polymeric Substances (EPS) Controlling Strategies for Performance Enhancement of Biological Wastewater Treatments: An Overview. Chemosphere.

[53] Na S.H., Shon H.K., Kim J.H. (2010). Minimization of Excess Sludge and Cryptic Growth of Microorganisms by Alkaline Treatment of Activated Sludge. Korean J. Chem. Eng..

[54] Henderson R.K., Baker A., Murphy K.R., Hambly A., Stuetz R.M., Khan S.J. (2009). Fluorescence as a Potential Monitoring Tool for Recycled Water Systems: A Review. Water Res..

[55] Liang W., Yang B., Bin L., Hu Y., Fan D., Chen W., Li P., Tang B. (2024). Intensifying the Simultaneous Removal of Nitrogen and Phosphorus of an Integrated Aerobic Granular Sludge–Membrane Bioreactor by *Acinetobacter junii*. Bioresour. Technol..

[56] Nsabimana E., Belan A., Bohatier J. (2000). Analysis at the Genomospecies Level of Microbial Populations Changes in Activated Sludge: The Case of Aeromonas. Water Res..

[57] Schulze R., Spring S., Amann R., Huber I., Ludwig W., Schleifer K.-H., Kämpfer P. (1999). Genotypic Diversity of *Acidovorax* Strains Isolated from Activated Sludge and Description of *Acidovorax defluvii* sp. nov. Syst. Appl. Microbiol..

[58] Huang G., Wang X., Pan D., Yang G., Zhong R., Niu R., Xia B., Cheng K., Liu T., Li X. (2023). Cadmium Immobilization during Nitrate-Reducing Fe(II) Oxidation by *Acidovorax* sp. BoFeN1: Contribution of Bacterial Cells and Secondary Minerals. Chem. Geol..

[59] Ge Q., Yue X., Wang G. (2015). Simultaneous Heterotrophic Nitrification and Aerobic Denitrification at High Initial Phenol Concentration by Isolated Bacterium *Diaphorobacter* sp. PD-7. Chin. J. Chem. Eng..

[60] Wang Y., Zhou Z., Zhang W., Guo J., Li N., Zhang Y., Gong D., Lyu Y. (2024). Metabolic Mechanism of Cr(VI) Pollution Remediation by *Alicycliphilus denitrificans* Ylb10. Sci. Total Environ..

[61] Zhang J., Dong Y., Wang Q., Xu D., Lv L., Gao W., Sun L., Zhang G., Ren Z. (2023). Effects of Ultrasonic Lysis Frequency on Sludge Lysis–Cryptic Growth: Sludge Reduction, Microbial Community, and Metabolism. Chem. Eng. J..

[62] Zhang M., Zhao B., Yan Y., Cheng Z., Li Z., Han L., Sun Y., Zheng Y., Xia Y. (2024). Comamonas-Dominant Microbial Community in Carbon-Poor Aquitard Sediments Revealed by Metagenomic-Based Growth Rate Investigation. Sci. Total Environ..

[63] Li X., Zhao X., Chen Z., Shen J., Jiang F., Wang X., Kang J. (2020). Isolation of Oxytetracycline-Degrading Bacteria and Its Application in Improving the Removal Performance of Aerobic Granular Sludge. J. Environ. Manag..

[64] Xu X., Cao D., Wang Z., Liu J., Gao J., Sanchuan M., Wang Z. (2019). Study on Ultrasonic Treatment for Municipal Sludge. Ultrason. Sonochem..

[65] Sun H., Wu Q., Yu P., Zhang L., Ye L., Zhang X.-X., Ren H. (2017). Denitrification Using Excess Activated Sludge as Carbon Source: Performance and Microbial Community Dynamics. Bioresour. Technol..

[66] Xu Z., Dai X., Chai X. (2018). Effect of Different Carbon Sources on Denitrification Performance, Microbial Community Structure and Denitrification Genes. Sci. Total Environ..

[67] Li S., Wang S., Ji G. (2022). Influences of Carbon Sources on N_2_O Production during Denitrification in Freshwaters: Activity, Isotopes and Functional Microbes. Water Res..

[68] Zhao K., Zhao S., Song G., Lu C., Liu R., Hu C., Qu J. (2023). Ultrasonication-Enhanced Biogas Production in Anaerobic Digestion of Waste Active Sludge: A Pilot-Scale Investigation. Resour. Conserv. Recycl..

[69] Lin Y.L., Chen S.T., Zheng N.Y., Wang H.C. (2023). Green Sludge Dewatering and Recycling Technology for Generating Renewable Energy and Liquid Nutrients: Bench- and Pilot-Scale Studies. Energy.

