# Supplementary material for: Ultrasonic Cavitation Transforms Organic Matter to Achieve Reduction of Excess Sludge and Recycling of Carbon Sources
Source: Toxics. 2025 Oct 31;13(11):941. doi: 10.3390/toxics13110941 (PMC12656604; doi:10.3390/toxics13110941)
Supplement: Supplementary file 1 [file toxics-13-00941-s001.zip › toxics-3928639-supplementary.pdf]

Supporting Information

# Ultrasonic cavitation transforms organic matter to achieve reduction in excess sludge and recycling of carbon sources

## Text S1. The synthesis of MNPs and the functionalization of biological sludge

A total of 5 mL of MNPs (10 mg/mL) was mixed with 45 mL of polyallylamine hydrochloride solution (10 mg/mL), and then stabilized in an ultrasonic water bath with a frequency of 40 kHz and an output energy of 125 w for 60 min. After centrifugation at 4000 rpm for 15 min, the pellet was resuspended in 50 mL deionized water and dispersed by shaking. Finally, filtered the solution through a 0.22 μm filter and prepared for bacterial functionalization.

First, 50 mL of bio-sludge was centrifuged at 3000 rpm for 10 min, collecting bacterial particles and resuspending them in the same volume of deionized water. Then, the cell suspension was ultrasonically mixed with 50 mL of polyallylamine hydrochloride stabilized MNPs solution and oscillated at room temperature at 150 r/min for 20 min. Next, the MNPs-functionalized bacteria were separated from the aqueous phase using permanent magnets and then suspended again in deionized water. The washing step was repeated three times to remove those bacteria that were not functionalized by MNPs. Subsequently, the MNP-functionalized bacteria were resuspended in 50 mL of deionized water.

**Text S2. Extraction of TB-EPS**

First, centrifuge 50 mL of sludge sample at 4000 rpm for 10 min and decant the supernatant. Next, resuspend the sludge particles in the tube with 0.05% NaCl solution heated to 70°C, shake for 5 min, then centrifuge again at 4000 rpm for 15 min to separate the solid from the supernatant. After pouring off the supernatant, resuspend the remaining sludge in a 0.05% NaCl solution to a volume of 50 mL, shake for 5 min, and heat the sludge suspension in a 60°C water bath for 20 min. Subsequently, centrifuge the sludge mixture at 4000 rpm for 20 min and collect the supernatant as TB-EPS.

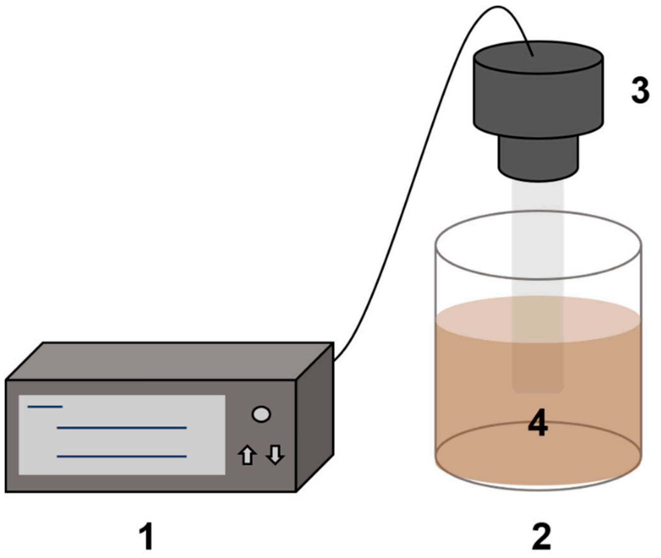


**Figure S1**. Schematic diagram of the ultrasonic cavitation experimental device: (1) Ultrasonic generator; (2) Reaction tank; (3) Ultrasonic transducer; (4) Ultrasonic probe.

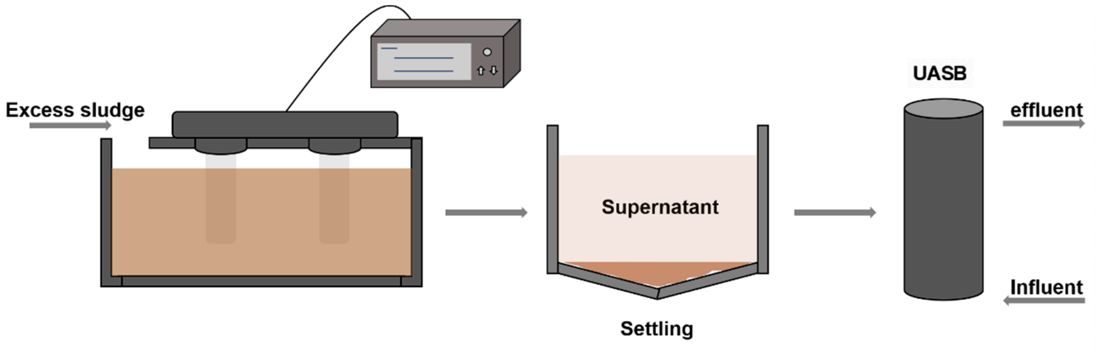


**Figure S2**. Schematic diagram of pilot-scale ultrasonic cavitation experiment process.

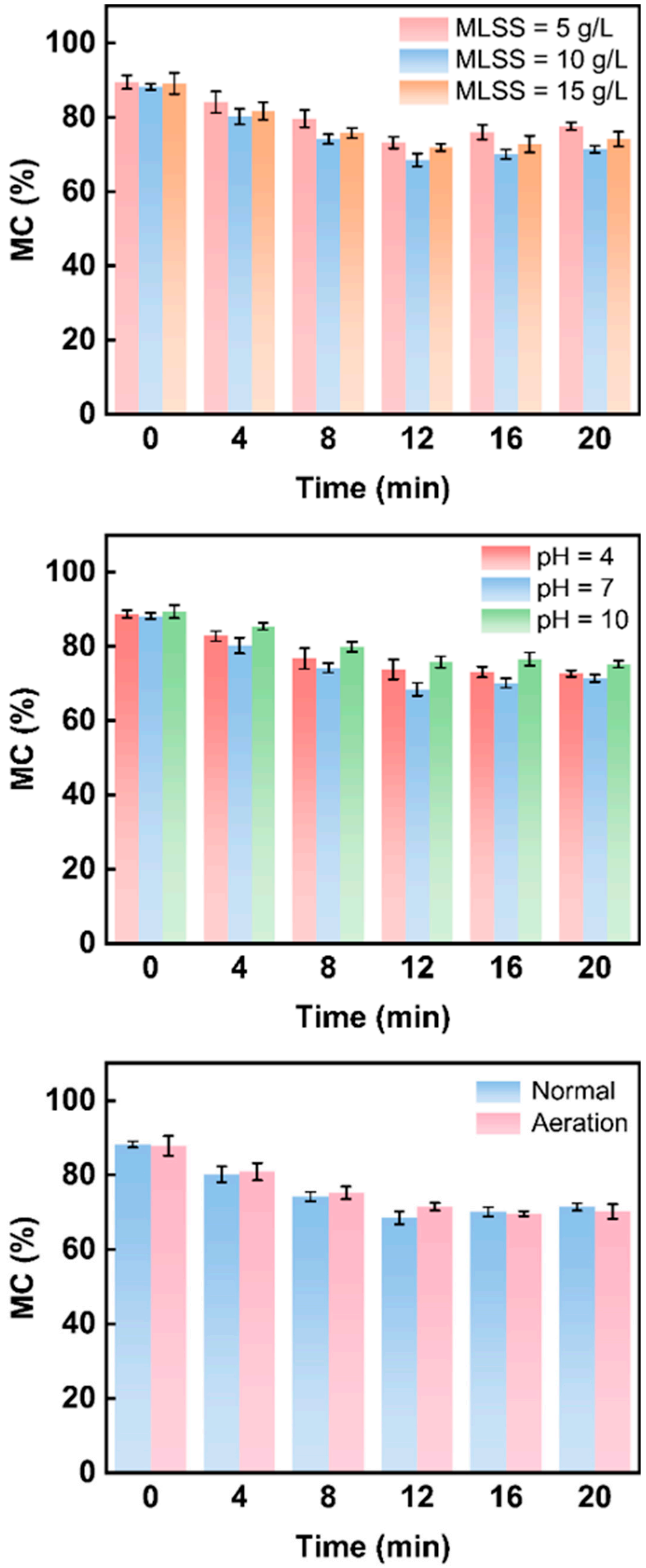


**Figure S3**. Changes in MC under different conditions.

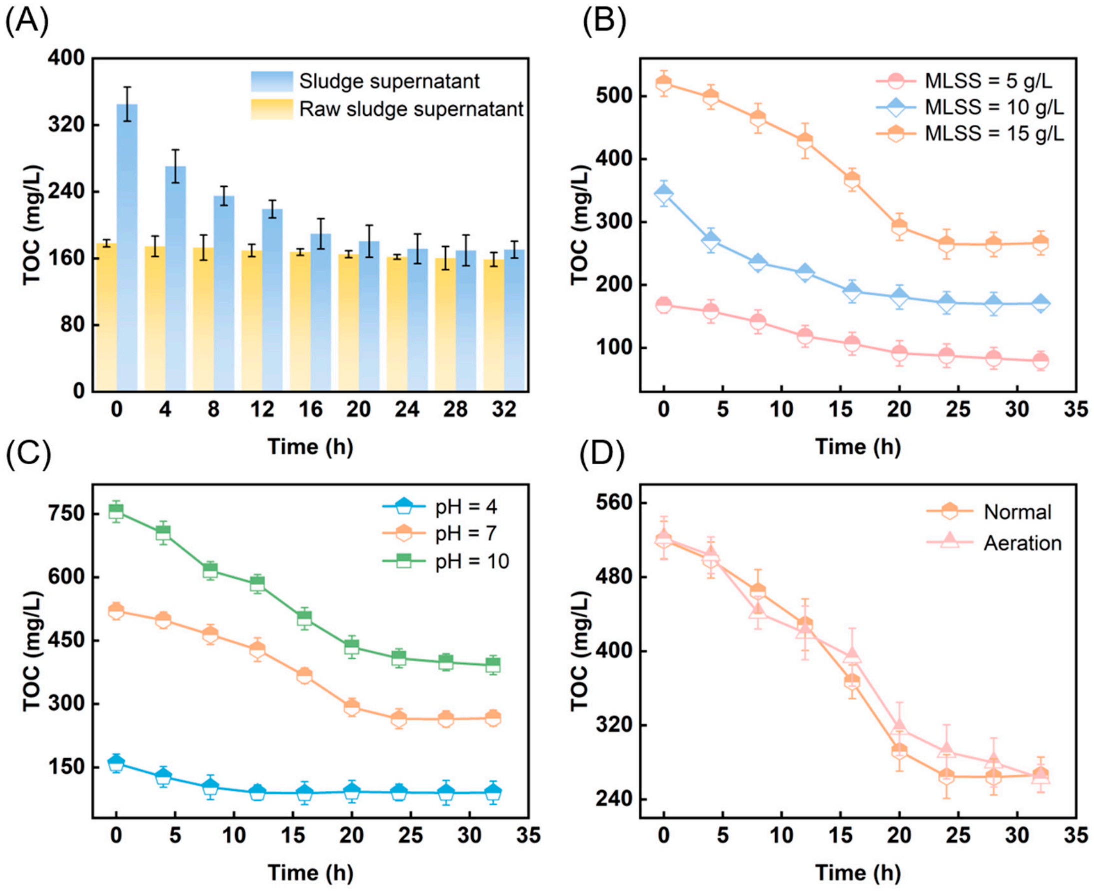


**Figure S4**. Changes in TOC concentration. (A) Feasibility of using sludge supernatant as a carbon source; (B) Initial sludge concentrations; (C) pH values; (D) Aeration condition.

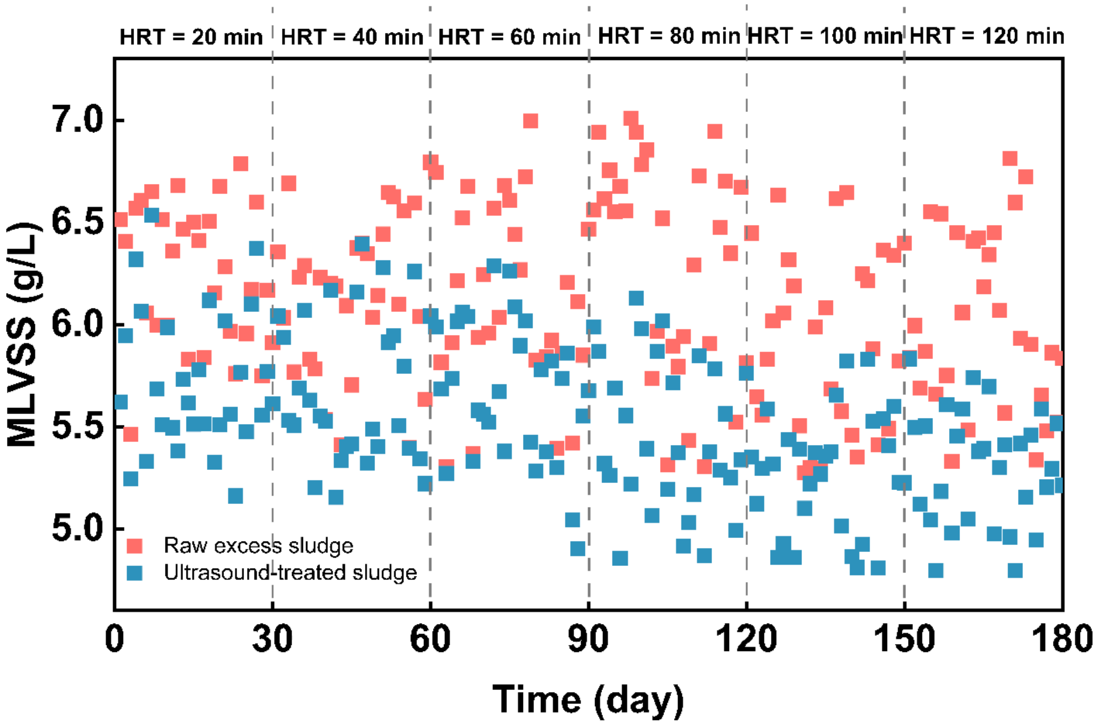


**Figure S5**. The effect of pilot-scale ultrasonic cavitation treatment on MLVSS.

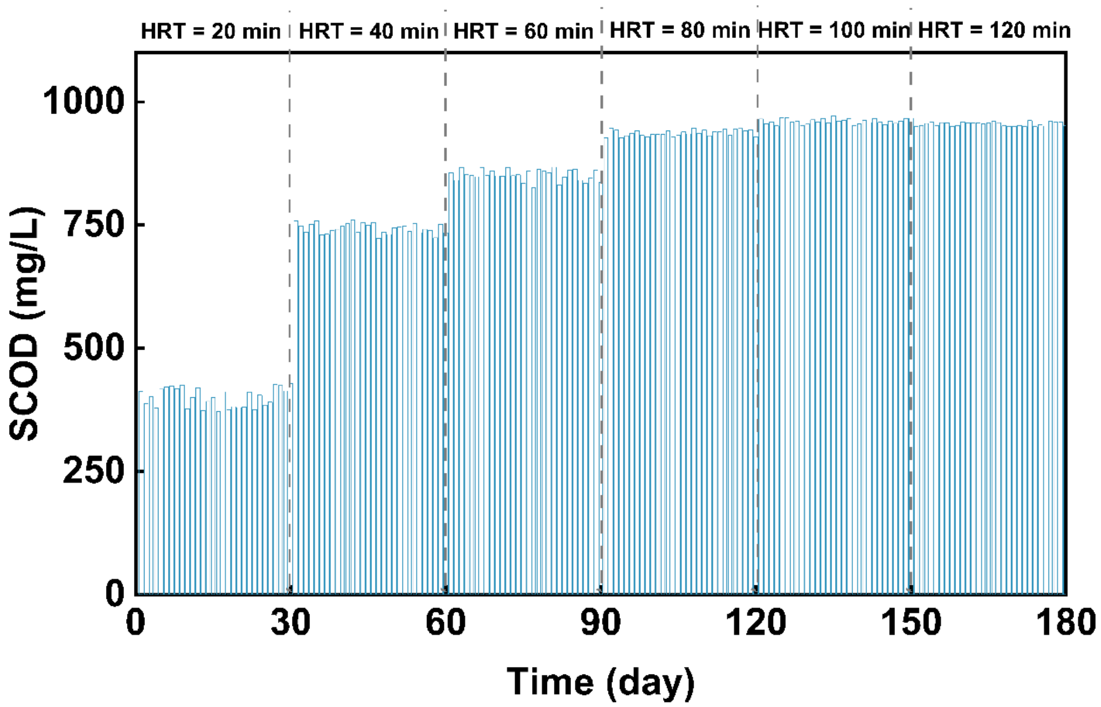


**Figure S6**. The effect of pilot-scale ultrasonic cavitation treatment on SCOD.

**Table S1.** The basic characteristics of the raw excess sludge.

| Parameter | Value |
|---|---|
| Moisture content (MC) [ %] | 98.2 ± 0.8 |
| Mixed liquid suspended solids (MLSS) [g/L] | 8.78 ± 0.4 |
| Soluble chemical oxygen demand (SCOD) [g/L] | 276 ± 8.2 |
| pH | 6.7 ± 0.3 |
| Temperature [℃] | 16.8 ± 1.2 |

**Table S2.** Excitation and emitting (Ex/Em) wavelengths of fluorescence region.

| Region | Substance | Ex [nm] | Em [nm] |
|---|---|---|---|
| I | Tyrosine-like protein | 200-250 | 200-330 |
| II | Tryptophan-like protein | 200-250 | 330-380 |
| III | Fulvic acid-like organics | 200-250 | 380-500 |
| IV | Soluble microbial by-product | 250-400 | 200-380 |
| V | Humic acid-like organics | 250-400 | 380-500 |

**Table S3.** Host bacteria carrying organic degradation functional genes in sample 1.

| Bin ID | Functional genes |
|---|---|
| bin.1 | *AA1, AA1_1, AA1_2, AA1_3, AA3_1, CBM48, CBM50, CE1, CE11, CE4, CE7, CE8, CE9, GH1, GH102, GH109, GH13, GH13_1, GH13_10, GH13_12, GH13_13, GH13_14, GH13_15, GH13_16, GH13_17, GH13_18, GH13_19, GH13_2, GH13_20, GH13_21, GH13_23, GH13_28, GH13_29, GH13_30, GH13_31, GH13_32, GH13_35, GH13_36, GH13_37, GH13_4, GH13_40, GH13_41, GH13_42, GH13_5, GH13_6, GH13_9, GH142, GH153, GH2, GH23, GH3, GH37, GH5, GH77, GH78, GH94, GT12, GT19, GT21, GT26, GT27, GT2_Glyco_tranf_2_2, GT2_Glyco_tranf_2_3, GT2_Glycos_transf_2, GT35, GT4, GT41, GT45, GT5, GT51, GT56, GT81, GT9* |
| bin.2 | *AA1, AA1_1, AA1_2, AA1_3, AA3, AA3_1, AA3_2, AA3_3, AA3_4, AA4, AA5, AA5_2, AA6, AA7, CBM50, CE1, CE11, CE12, CE19, CE3, CE4, CE7, CE9, GH102, GH103, GH109, GH114, GH135, GH166, GH23, GH3, GH74, GT104, GT19, GT28, GT2_Glyco_tranf_2_3, GT2_Glycos_transf_2, GT30, GT32, GT4, GT41, GT5, GT51, GT81, GT83, PL22, PL22_1, PL31, PL6, PL9, PL9_1, PL9_3* |
| bin.3 | *AA1, AA1_1, AA1_2, AA1_3, AA2, AA3, AA3_1, AA3_2, AA3_4, AA4, AA7, CBM50, CBM9, CE1, CE11, CE14, CE3, CE7, CE9, GH105, GH109, GH120, GH123, GH151, GH171, GH20, GH23, GH29, GH3, GH33, GH74, GH76, GH91, GH93, GT1, GT112, GT19, GT21, GT27, GT28, GT2_Chitin_synth_2, GT2_Glyco_tranf_2_2, GT2_Glyco_tranf_2_3, GT2_Glyco_trans_2_3, GT2_Glycos_transf_2, GT30, GT4, GT41, GT45, GT5, GT51, GT7, GT81, GT9, PL1, PL22, PL22_1, PL22_2, PL31, PL41, PL6, PL6_2, PL9_2* |
| bin.4 | *AA1, AA1_1, AA1_2, AA1_3, AA2, AA3, AA3_1, AA3_2, AA3_3, AA3_4, AA4, AA7, CBM20, CBM32, CBM48, CBM50, CBM67, CBM71, CBM8, CBM9, CE1, CE14, CE19, CE3, CE4, CE7, CE8, CE9, GH104, GH106, GH109, GH117, GH121, GH123, GH13, GH13_1, GH13_10, GH13_11, GH13_12, GH13_13, GH13_14, GH13_15, GH13_16, GH13_17, GH13_18, GH13_19, GH13_2, GH13_20, GH13_21, GH13_22, GH13_23, GH13_24, GH13_26, GH13_27, GH13_28, GH13_29, GH13_3, GH13_30, GH13_31, GH13_32, GH13_34, GH13_35, GH13_36, GH13_37, GH13_38, GH13_39, GH13_4, GH13_40, GH13_41, GH13_42, GH13_5, GH13_6, GH13_7, GH13_8, GH13_9, GH142, GH147, GH151, GH156, GH158, GH16, GH16_1, GH16_10, GH16_13, GH16_14, GH16_16, GH16_17, GH16_18, GH16_19, GH16_2, GH16_20, GH16_21, GH16_22, GH16_23, GH16_24, GH16_25, GH16_27,* |

| | |
|---|---|
| | *GH16_3, GH16_4, GH16_5, GH16_6, GH16_7, GH16_8, GH16_9, GH171, GH18, GH2, GH20, GH23, GH29, GH3, GH32, GH33, GH37, GH43, GH43_1, GH43_10, GH43_11, GH43_12, GH43_13, GH43_14, GH43_15, GH43_16, GH43_17, GH43_18, GH43_19, GH43_2, GH43_22, GH43_23, GH43_24, GH43_25, GH43_26, GH43_27, GH43_28, GH43_29, GH43_3, GH43_30, GH43_31, GH43_32, GH43_33, GH43_34, GH43_35, GH43_37, GH43_4, GH43_5, GH43_6, GH43_7, GH43_8, GH43_9, GH53, GH74, GH76, GH77, GH78, GH93, GH94, GH97, GT1, GT19, GT21, GT27, GT28, GT2_Chitin_synth_2, GT2_Glyco_tranf_2_2, GT2_Glyco_tranf_2_3, GT2_Glyco_tranf_2_4, GT2_Glyco_trans_2_3, GT2_Glycos_transf_2, GT39, GT4, GT41, GT5, GT51, GT7, GT81, GT83, GT9, PL22, PL22_1, PL41, PL4_1, PL9, PL9_1, PL9_2, PL9_3* |
| bin.5 | *AA2, AA3, AA3_1, AA3_2, AA3_4, AA4, AA5, AA7, CBM11, CBM16, CBM23, CBM32, CBM35, CBM47, CBM48, CBM50, CBM57, CBM6, CBM62, CBM9, CE14, CE4, CE7, CE9, GH100, GH102, GH109, GH119, GH133, GH13_25, GH142, GH144, GH171, GH23, GH3, GH57, GH76, GH77, GH78, GH94, GT1, GT105, GT19, GT20, GT21, GT26, GT27, GT28, GT2_Glyco_tranf_2_2, GT2_Glyco_tranf_2_3, GT2_Glyco_tranf_2_4, GT2_Glyco_trans_2_3, GT2_Glycos_transf_2, GT35, GT39, GT4, GT41, GT5, GT51, GT81, GT83, GT84, GT9, GT94, PL22, PL22_1* |
| bin.6 | *AA1, AA12, AA1_1, AA1_2, AA1_3, AA2, AA3, AA3_1, AA3_2, AA3_3, AA3_4, AA4, AA6, AA7, CBM32, CBM50, CE1, CE12, CE19, CE3, CE4, CE7, CE9, GH1, GH102, GH103, GH108, GH117, GH171, GH23, GH25, GH3, GH39, GH4, GH42, GH43, GH43_10, GH43_11, GH43_12, GH43_13, GH43_14, GH43_15, GH43_16, GH43_17, GH43_18, GH43_19, GH43_2, GH43_22, GH43_23, GH43_24, GH43_26, GH43_27, GH43_28, GH43_29, GH43_3, GH43_30, GH43_31, GH43_32, GH43_33, GH43_34, GH43_36, GH43_4, GH43_5, GH43_8, GH43_9, GH5, GH5_29, GH65, GT1, GT19, GT22, GT27, GT28, GT2_Glyco_tranf_2_2, GT2_Glyco_tranf_2_3, GT2_Glyco_trans_2_3, GT2_Glycos_transf_2, GT4, GT41, GT5, GT51, GT81, PL1, PL12, PL12_3, PL17, PL17_2, PL1_2, PL22, PL22_1, PL22_2, PL31, PL39, PL41, PL6, PL6_1, PL6_2, PL6_3, PL9, PL9_1, PL9_3* |
| bin.7 | *AA3, AA3_1, AA3_2, AA3_3, AA4, AA6, AA7, CBM48, CBM50, CBM9, CE1, CE11, CE12, CE16, CE17, CE19, CE3, CE4, CE7, CE9, GH102, GH103, GH119, GH13, GH13_1, GH13_10, GH13_11, GH13_12, GH13_13, GH13_14, GH13_16, GH13_17, GH13_18, GH13_19, GH13_2, GH13_20, GH13_21, GH13_22, GH13_23, GH13_26, GH13_27, GH13_28, GH13_29, GH13_3, GH13_30, GH13_31, GH13_32, GH13_34, GH13_35, GH13_36, GH13_37, GH13_38, GH13_39, GH13_4, GH13_40, GH13_41, GH13_42, GH13_5, GH13_6, GH13_7, GH13_8, GH13_9, GH15, GH23, GH3, GH78, GH97, GT1, GT19, GT20, GT21, GT27, GT28, GT2_Glyco_tranf_2_2, GT2_Glyco_tranf_2_3, GT2_Glyco_tranf_2_4, GT2_Glycos_transf_2, GT30, GT4, GT41, GT5, GT51, GT81, GT83, GT9, PL1, PL10, PL10_1, PL10_2, PL10_3, PL1_2, PL22* |

| | |
|---|---|
| bin.10 | *AA2, AA3, AA3_1, AA3_2, AA3_3, AA3_4, AA4, AA7, CBM16, CBM20, CBM32, CBM40, CBM47, CBM48, CBM50, CBM62, CBM66, CBM9, CE1, CE11, CE12, CE14, CE17, CE19, CE2, CE3, CE4, CE6, CE7, CE8, CE9, GH105, GH109, GH120, GH13, GH136, GH139, GH13_1, GH13_10, GH13_11, GH13_12, GH13_13, GH13_14, GH13_15, GH13_16, GH13_17, GH13_18, GH13_19, GH13_2, GH13_20, GH13_21, GH13_22, GH13_23, GH13_26, GH13_27, GH13_28, GH13_29, GH13_3, GH13_30, GH13_31, GH13_32, GH13_34, GH13_35, GH13_36, GH13_37, GH13_38, GH13_39, GH13_4, GH13_40, GH13_41, GH13_42, GH13_5, GH13_6, GH13_7, GH13_8, GH13_9, GH150, GH151, GH171, GH20, GH23, GH28, GH29, GH3, GH30, GH30_1, GH30_2, GH30_3, GH30_4, GH30_5, GH30_6, GH30_7, GH30_8, GH30_9, GH33, GH35, GH36, GH42, GH43, GH43_1, GH43_10, GH43_11, GH43_12, GH43_13, GH43_14, GH43_15, GH43_16, GH43_2, GH43_24, GH43_28, GH43_29, GH43_3, GH43_30, GH43_31, GH43_32, GH43_33, GH43_34, GH43_35, GH43_36, GH43_37, GH43_4, GH43_5, GH43_8, GH43_9, GH5, GH53, GH70, GH73, GH74, GH76, GH77, GH87, GH9, GH91, GH92, GH93, GH95, GH97, GT12, GT17, GT19, GT21, GT25, GT27, GT28, GT2_Glyco_tranf_2_2, GT2_Glyco_tranf_2_3, GT2_Glyco_tranf_2_4, GT2_Glyco_tranf_2_5, GT2_Glyco_trans_2_3, GT2_Glycos_transf_2, GT30, GT32, GT35, GT4, GT40, GT41, GT45, GT5, GT51, GT81, GT9, GT94, PL1, PL1_2, PL22, PL22_1, PL22_2, PL31* |
| bin.11 | *AA1, AA12, AA1_3, AA3, AA3_1, AA3_2, AA3_3, AA3_4, AA4, AA6, AA7, CBM48, CBM50, CBM67, CE1, CE11, CE14, CE19, CE3, CE4, CE5, CE7, CE9, GH103, GH109, GH119, GH13, GH136, GH13_1, GH13_10, GH13_11, GH13_12, GH13_13, GH13_14, GH13_16, GH13_17, GH13_20, GH13_21, GH13_23, GH13_26, GH13_28, GH13_3, GH13_30, GH13_31, GH13_32, GH13_36, GH13_37, GH13_4, GH13_40, GH13_41, GH13_8, GH13_9, GH15, GH17, GH171, GH23, GH3, GH33, GH37, GH5, GH51, GH53, GH57, GH5_12, GH5_22, GH5_27, GH5_28, GH5_29, GH5_39, GH5_43, GH5_5, GH74, GH77, GH78, GH93, GT1, GT105, GT19, GT20, GT21, GT27, GT28, GT2_Glyco_tranf_2_2, GT2_Glyco_tranf_2_3, GT2_Glyco_trans_2_3, GT2_Glycos_transf_2, GT30, GT35, GT4, GT41, GT5, GT51, GT7, GT81, GT83, GT9, PL22, PL22_1, PL31, PL6, PL6_1, PL6_2, PL9, PL9_1, PL9_2, PL9_3* |
| bin.12 | *AA1, AA12, AA1_1, AA1_2, AA1_3, AA3, AA3_1, AA3_2, AA3_3, AA3_4, AA4, AA5, AA5_2, AA6, AA7, CBM50, CBM9, CE1, CE11, CE16, CE3, CE4, CE7, CE9, GH102, GH23, GH33, GH39, GH74, GH77, GH93, GH99, GT1, GT102, GT105, GT112, GT19, GT21, GT27, GT28, GT2_Glyco_tranf_2_2, GT2_Glyco_tranf_2_3, GT2_Glyco_tranf_2_4, GT2_Glyco_tranf_2_5, GT2_Glyco_trans_2_3, GT2_Glycos_transf_2, GT30, GT4, GT40, GT41, GT45, GT5, GT51, GT8, GT81, GT83, GT9, PL22, PL22_1* |

| | |
|---|---|
| bin.14 | *AA1, AA12, AA1_1, AA1_2, AA1_3, AA3, AA3_1, AA3_2, AA3_3, AA3_4, AA7, CBM16, CBM20, CBM32, CBM35, CBM37, CBM40, CBM47, CBM48, CBM50, CBM66, CBM67, CBM88, CBM9, CE1, CE11, CE19, CE2, CE3, CE4, CE7, CE8, CE9, GH1, GH102, GH108, GH109, GH120, GH127, GH13, GH136, GH13_1, GH13_10, GH13_11, GH13_12, GH13_13, GH13_14, GH13_15, GH13_16, GH13_17, GH13_18, GH13_19, GH13_2, GH13_20, GH13_21, GH13_22, GH13_23, GH13_26, GH13_27, GH13_28, GH13_29, GH13_3, GH13_30, GH13_31, GH13_32, GH13_34, GH13_35, GH13_36, GH13_37, GH13_38, GH13_39, GH13_4, GH13_40, GH13_41, GH13_42, GH13_5, GH13_6, GH13_7, GH13_8, GH13_9, GH140, GH144, GH146, GH149, GH151, GH158, GH16, GH161, GH163, GH16_13, GH16_14, GH16_2, GH16_21, GH16_24, GH16_3, GH16_4, GH16_5, GH16_6, GH16_7, GH16_8, GH16_9, GH17, GH171, GH18, GH2, GH20, GH23, GH27, GH29, GH3, GH30, GH30_1, GH30_2, GH30_3, GH30_4, GH30_5, GH30_6, GH30_7, GH30_8, GH30_9, GH31, GH32, GH33, GH36, GH37, GH43, GH43_1, GH43_28, GH43_29, GH43_3, GH43_30, GH43_31, GH43_33, GH43_34, GH51, GH53, GH57, GH5_42, GH63, GH65, GH70, GH73, GH74, GH76, GH77, GH78, GH87, GH93, GH95, GH97, GT105, GT19, GT21, GT22, GT27, GT28, GT2_Cellulose_synt, GT2_Glyco_tranf_2_2, GT2_Glyco_tranf_2_3, GT2_Glyco_tranf_2_4, GT2_Glyco_trans_2_3, GT2_Glycos_transf_2, GT35, GT39, GT4, GT40, GT41, GT5, GT51, GT81, GT83, GT9, GT94, PL11, PL22, PL22_1, PL22_2, PL31, PL41, PL4_1, PL6, PL6_1, PL6_2, PL9, PL9_1, PL9_2, PL9_3, PL9_4, cohesin* |
| bin.16 | *AA2, AA3, AA3_1, AA3_2, AA3_3, AA4, AA5, AA5_1, AA5_2, AA7, CBM48, CBM50, CBM67, CBM9, CE1, CE11, CE12, CE16, CE19, CE3, CE4, CE7, CE8, CE9, GH1, GH103, GH105, GH109, GH13, GH130, GH13_1, GH13_10, GH13_11, GH13_12, GH13_13, GH13_14, GH13_16, GH13_17, GH13_18, GH13_19, GH13_2, GH13_20, GH13_21, GH13_22, GH13_23, GH13_26, GH13_27, GH13_28, GH13_29, GH13_3, GH13_30, GH13_31, GH13_32, GH13_34, GH13_35, GH13_36, GH13_37, GH13_38, GH13_39, GH13_4, GH13_40, GH13_41, GH13_42, GH13_5, GH13_6, GH13_7, GH13_8, GH13_9, GH154, GH16, GH16_21, GH18, GH20, GH23, GH28, GH3, GH30, GH30_1, GH30_2, GH30_3, GH30_4, GH30_5, GH30_6, GH30_7, GH30_8, GH30_9, GH32, GH33, GH35, GH42, GH43, GH43_18, GH43_19, GH43_20, GH43_22, GH43_23, GH43_3, GH43_30, GH43_34, GH43_8, GH5, GH55, GH59, GH73, GH74, GH77, GH93, GT1, GT102, GT104, GT12, GT19, GT21, GT23, GT26, GT27, GT28, GT2_Glyco_tranf_2_2, GT2_Glyco_tranf_2_3, GT2_Glyco_tranf_2_4, GT2_Glycos_transf_2, GT30, GT35, GT4, GT40, GT41, GT45, GT5, GT51, GT81, GT83, GT9, PL1, PL11, PL11_1, PL11_2, PL12, PL12_1, PL12_3, PL15, PL15_1, PL15_2, PL1_1, PL1_10, PL1_11, PL1_12, PL1_13, PL1_3, PL1_4, PL1_5, PL1_6, PL1_7, PL1_8, PL1_9, PL22, PL22_1, PL22_2, PL26, PL31, PL39, PL41, PL6, PL6_1, PL9, PL9_2, dockerin* |

| | |
|---|---|
| bin.17 | *AA12, AA3, AA3_1, AA3_2, AA3_4, AA5, AA7, CBM16, CBM20, CBM22, CBM30, CBM32, CBM35, CBM37, CBM40, CBM47, CBM48, CBM50, CBM56, CBM6, CBM61, CBM62, CBM70, CBM77, CBM8, CBM88, CBM9, CE1, CE11, CE12, CE14, CE19, CE2, CE3, CE4, CE6, CE7, CE8, CE9, GH100, GH105, GH109, GH110, GH119, GH125, GH13, GH130, GH133, GH138, GH13_1, GH13_10, GH13_11, GH13_12, GH13_13, GH13_14, GH13_15, GH13_16, GH13_17, GH13_19, GH13_2, GH13_20, GH13_21, GH13_22, GH13_23, GH13_26, GH13_27, GH13_28, GH13_29, GH13_3, GH13_30, GH13_31, GH13_32, GH13_34, GH13_35, GH13_36, GH13_37, GH13_38, GH13_39, GH13_4, GH13_40, GH13_41, GH13_42, GH13_5, GH13_6, GH13_7, GH13_8, GH13_9, GH144, GH149, GH15, GH158, GH16, GH161, GH16_1, GH16_10, GH16_11, GH16_12, GH16_13, GH16_14, GH16_17, GH16_18, GH16_19, GH16_2, GH16_20, GH16_21, GH16_23, GH16_24, GH16_25, GH16_27, GH16_3, GH16_4, GH16_5, GH16_6, GH16_7, GH16_8, GH16_9, GH17, GH171, GH18, GH19, GH2, GH20, GH23, GH24, GH25, GH26, GH28, GH29, GH3, GH30, GH30_1, GH30_2, GH30_3, GH30_4, GH30_5, GH30_6, GH30_7, GH30_8, GH30_9, GH37, GH43, GH43_1, GH43_10, GH43_11, GH43_12, GH43_13, GH43_14, GH43_15, GH43_16, GH43_17, GH43_18, GH43_19, GH43_2, GH43_22, GH43_24, GH43_26, GH43_28, GH43_29, GH43_3, GH43_30, GH43_31, GH43_32, GH43_33, GH43_34, GH43_36, GH43_37, GH43_4, GH43_5, GH43_8, GH43_9, GH5, GH59, GH5_1, GH5_14, GH5_15, GH5_19, GH5_2, GH5_21, GH5_22, GH5_23, GH5_24, GH5_25, GH5_26, GH5_27, GH5_28, GH5_29, GH5_30, GH5_31, GH5_36, GH5_37, GH5_38, GH5_39, GH5_4, GH5_40, GH5_41, GH5_42, GH5_44, GH5_45, GH5_46, GH5_47, GH5_48, GH5_49, GH5_5, GH5_50, GH5_51, GH5_52, GH5_54, GH5_55, GH5_7, GH5_8, GH5_9, GH63, GH70, GH72, GH73, GH74, GH76, GH77, GH78, GH87, GH92, GH97, GT101, GT113, GT12, GT14, GT19, GT21, GT27, GT28, GT2_Chitin_synth_2, GT2_Glyco_tranf_2_2, GT2_Glyco_tranf_2_3, GT2_Glyco_tranf_2_4, GT2_Glyco_tranf_2_5, GT2_Glyco_trans_2_3, GT2_Glycos_transf_2, GT30, GT33, GT35, GT4, GT41, GT45, GT5, GT51, GT74, GT81, GT87, GT89, GT9, GT90, PL1, PL10, PL10_1, PL10_2, PL10_3, PL1_1, PL1_10, PL1_11, PL1_12, PL1_13, PL1_2, PL1_3, PL1_4, PL1_5, PL1_6, PL1_7, PL1_8, PL1_9, PL22, PL22_1, PL32, PL9_1, PL9_2* |
| bin.18 | *AA2, AA3, AA3_1, AA3_2, AA3_3, AA3_4, AA4, AA6, AA7, CBM50, CE1, CE11, CE19, CE3, CE4, CE7, CE9, GH102, GH103, GH108, GH109, GH113, GH18, GH23, GH25, GH3, GH33, GH74, GH93, GT1, GT107, GT14, GT19, GT21, GT23, GT25, GT27, GT28, GT29, GT2_Chitin_synth_2, GT2_Glyco_tranf_2_2, GT2_Glyco_tranf_2_3, GT2_Glyco_tranf_2_4, GT2_Glyco_trans_2_3, GT2_Glycos_transf_2, GT30, GT39, GT4, GT41, GT45, GT5, GT51, GT81, GT83, GT92, GT99, PL12, PL12_3, PL22, PL31, PL41, PL6, PL6_1* |

| | |
|---|---|
| bin.19 | *AA3, AA3_1, AA3_2, AA3_3, AA4, AA6, AA7, CBM48, CBM50, CE1, CE11, CE12, CE16, CE3, CE4, CE7, CE9, GH102, GH103, GH108, GH109, GH13, GH13_1, GH13_10, GH13_11, GH13_12, GH13_13, GH13_14, GH13_16, GH13_17, GH13_19, GH13_2, GH13_20, GH13_21, GH13_23, GH13_26, GH13_28, GH13_29, GH13_3, GH13_30, GH13_31, GH13_32, GH13_36, GH13_37, GH13_38, GH13_39, GH13_41, GH13_42, GH13_5, GH13_6, GH13_8, GH13_9, GH15, GH2, GH23, GH3, GH63, GH73, GH77, GH78, GT1, GT10, GT104, GT19, GT20, GT21, GT27, GT28, GT2_Glyco_tranf_2_3, GT2_Glyco_trans_2_3, GT2_Glycos_transf_2, GT30, GT35, GT39, GT4, GT41, GT5, GT51, GT81, GT83, PL12, PL12_2, PL15, PL15_1, PL17, PL21, PL21_1, PL22, PL31, PL39, PL41* |
| bin.20 | *AA1, AA1_1, AA1_2, AA1_3, AA2, AA3, AA3_1, AA3_2, AA3_3, AA4, AA6, AA7, CBM16, CBM22, CBM50, CBM61, CE1, CE19, CE4, CE7, CE9, GH102, GH103, GH109, GH167, GH171, GH19, GH23, GH3, GT1, GT104, GT19, GT25, GT27, GT28, GT2_Glyco_tranf_2_3, GT2_Glyco_tranf_2_4, GT2_Glyco_trans_2_3, GT2_Glycos_transf_2, GT30, GT4, GT40, GT41, GT51, GT81, GT83, PL11, PL11_2, PL22, PL22_1* |
| bin.21 | *AA1, AA12, AA1_1, AA1_2, AA1_3, AA3, AA3_1, AA3_2, AA3_3, AA4, AA6, AA7, CBM13, CBM50, CE1, CE12, CE19, CE3, CE4, CE7, CE9, GH102, GH103, GH125, GH13, GH13_1, GH13_10, GH13_11, GH13_12, GH13_13, GH13_14, GH13_15, GH13_16, GH13_17, GH13_18, GH13_19, GH13_2, GH13_20, GH13_21, GH13_22, GH13_23, GH13_24, GH13_26, GH13_27, GH13_28, GH13_29, GH13_3, GH13_30, GH13_31, GH13_32, GH13_34, GH13_35, GH13_36, GH13_37, GH13_38, GH13_39, GH13_4, GH13_40, GH13_41, GH13_42, GH13_5, GH13_6, GH13_7, GH13_8, GH13_9, GH16, GH16_1, GH16_10, GH16_11, GH16_12, GH16_13, GH16_14, GH16_17, GH16_2, GH16_21, GH16_24, GH16_26, GH16_3, GH16_4, GH16_5, GH16_6, GH16_7, GH16_8, GH16_9, GH171, GH18, GH23, GH24, GH25, GH28, GH3, GH30, GH30_1, GH30_2, GH30_3, GH30_4, GH30_5, GH30_6, GH30_7, GH30_8, GH30_9, GH4, GH43, GH59, GH68, GH87, GH97, GT1, GT21, GT27, GT28, GT2_Glyco_tranf_2_2, GT2_Glyco_tranf_2_3, GT2_Glyco_trans_2_3, GT2_Glycos_transf_2, GT39, GT4, GT41, GT45, GT5, GT51, GT66, GT81, GT83, PL1, PL12, PL12_3, PL22, PL22_1, PL22_2, PL31, PL6_2, PL9, PL9_1, PL9_2, PL9_4* |
| bin.23 | *AA1, AA12, AA1_1, AA1_2, AA1_3, AA3, AA3_1, AA3_2, AA3_3, AA3_4, AA4, AA5, AA5_1, AA5_2, AA6, AA7, CBM16, CBM48, CBM50, CBM61, CE1, CE11, CE19, CE4, CE5, CE7, CE9, GH100, GH103, GH105, GH13, GH133, GH13_1, GH13_10, GH13_11, GH13_12, GH13_13, GH13_14, GH13_16, GH13_17, GH13_19, GH13_2, GH13_20, GH13_21, GH13_23, GH13_25, GH13_26, GH13_28, GH13_29, GH13_3, GH13_30, GH13_31, GH13_32, GH13_36, GH13_37, GH13_38, GH13_39, GH13_4, GH13_40, GH13_41, GH13_42, GH13_5, GH13_6, GH13_8, GH13_9, GH15, GH23, GH3, GH63, GH73, GH76, GH77, GH78,* |

| | |
|---|---|
| | *GH94, GT100, GT105, GT13, GT19, GT20, GT21, GT27, GT28, GT2_Glyco_tranf_2_2, GT2_Glyco_tranf_2_3, GT2_Glyco_tranf_2_4, GT2_Glyco_trans_2_3, GT2_Glycos_transf_2, GT30, GT35, GT39, GT4, GT41, GT5, GT51, GT81, GT83, GT9, PL22* |
| bin.24 | *AA1, AA12, AA1_1, AA1_2, AA1_3, AA3, AA3_1, AA3_2, AA3_3, AA4, AA5, AA6, AA7, CBM50, CBM66, CE1, CE19, CE3, CE4, CE7, CE9, GH102, GH103, GH109, GH117, GH125, GH13, GH130, GH13_1, GH13_10, GH13_11, GH13_12, GH13_13, GH13_14, GH13_15, GH13_16, GH13_17, GH13_18, GH13_19, GH13_2, GH13_20, GH13_21, GH13_22, GH13_23, GH13_24, GH13_26, GH13_27, GH13_28, GH13_29, GH13_3, GH13_30, GH13_31, GH13_32, GH13_34, GH13_35, GH13_36, GH13_37, GH13_38, GH13_39, GH13_4, GH13_40, GH13_41, GH13_42, GH13_5, GH13_6, GH13_7, GH13_8, GH13_9, GH23, GH24, GH25, GH28, GH3, GH4, GH43, GH43_1, GH43_10, GH43_11, GH43_12, GH43_13, GH43_14, GH43_15, GH43_16, GH43_17, GH43_18, GH43_19, GH43_2, GH43_22, GH43_24, GH43_26, GH43_28, GH43_29, GH43_3, GH43_30, GH43_31, GH43_32, GH43_33, GH43_34, GH43_36, GH43_4, GH43_5, GH43_8, GH43_9, GH87, GH97, GT107, GT22, GT27, GT28, GT2_Glyco_tranf_2_3, GT2_Glyco_trans_2_3, GT2_Glycos_transf_2, GT39, GT4, GT41, GT5, GT51, GT81, GT83, PL1, PL12, PL12_3, PL1_2, PL22, PL22_1, PL31, PL9, PL9_1, PL9_2* |
| bin.31 | *AA12, AA3, AA3_1, AA3_2, AA3_3, AA3_4, AA4, AA5, AA6, AA7, CBM13, CBM50, CBM66, CE1, CE19, CE3, CE4, CE7, CE9, GH102, GH103, GH125, GH13, GH13_1, GH13_10, GH13_11, GH13_12, GH13_13, GH13_14, GH13_15, GH13_16, GH13_17, GH13_18, GH13_19, GH13_2, GH13_20, GH13_21, GH13_22, GH13_23, GH13_24, GH13_26, GH13_27, GH13_28, GH13_29, GH13_3, GH13_30, GH13_31, GH13_32, GH13_34, GH13_35, GH13_36, GH13_37, GH13_38, GH13_39, GH13_4, GH13_40, GH13_41, GH13_42, GH13_5, GH13_6, GH13_7, GH13_8, GH13_9, GH171, GH20, GH23, GH24, GH25, GH28, GH3, GH30, GH30_1, GH30_2, GH30_3, GH30_4, GH30_5, GH30_6, GH30_7, GH30_8, GH30_9, GH39, GH4, GH59, GH87, GH94, GH97, GT107, GT14, GT21, GT25, GT27, GT28, GT2_Glyco_tranf_2_2, GT2_Glyco_tranf_2_3, GT2_Glyco_trans_2_3, GT2_Glycos_transf_2, GT3, GT39, GT4, GT41, GT45, GT5, GT51, GT81, GT83, PL1, PL12, PL12_3, PL22, PL22_1, PL22_2, PL31, PL6_2, PL9, PL9_1, PL9_2* |
| bin.32 | *AA12, AA3, AA3_1, AA3_2, AA3_3, AA3_4, AA4, AA6, AA7, CBM35, CBM48, CBM50, CBM84, CE1, CE11, CE19, CE4, CE5, CE6, CE7, CE9, GH103, GH104, GH109, GH119, GH13, GH136, GH13_1, GH13_10, GH13_11, GH13_12, GH13_13, GH13_14, GH13_16, GH13_17, GH13_2, GH13_20, GH13_21, GH13_23, GH13_26, GH13_28, GH13_3, GH13_30, GH13_31, GH13_32, GH13_36, GH13_37, GH13_4, GH13_40, GH13_41, GH13_8, GH13_9, GH15, GH17, GH171, GH23, GH3, GH33, GH5, GH5_1,* |

| | |
|---|---|
| | *GH5_12, GH5_14, GH5_15, GH5_22, GH5_23, GH5_25, GH5_27, GH5_28, GH5_29, GH5_33, GH5_36, GH5_37, GH5_38, GH5_39, GH5_43, GH5_44, GH5_45, GH5_46, GH5_47, GH5_49, GH5_5, GH5_50, GH5_52, GH5_54, GH5_8, GH5_9, GH73, GH74, GH78, GH93, GT1, GT105, GT19, GT20, GT21, GT27, GT28, GT2_Glyco_tranf_2_2, GT2_Glyco_tranf_2_3, GT2_Glyco_trans_2_3, GT2_Glycos_transf_2, GT30, GT35, GT39, GT4, GT41, GT45, GT5, GT51, GT81, GT83, GT9, PL12, PL12_1, PL12_3, PL17, PL22, PL22_1, PL31, PL6, PL6_1, PL6_2, PL9, PL9_1, PL9_2, PL9_3* |
| bin.33 | *AA1, AA12, AA1_1, AA1_2, AA1_3, AA3, AA3_1, AA3_2, AA4, AA5, AA6, AA7, CBM32, CBM48, CBM50, CE1, CE11, CE14, CE19, CE3, CE4, CE7, CE9, GH103, GH109, GH119, GH121, GH13, GH13_1, GH13_10, GH13_11, GH13_12, GH13_13, GH13_14, GH13_16, GH13_17, GH13_18, GH13_19, GH13_2, GH13_20, GH13_21, GH13_22, GH13_23, GH13_26, GH13_27, GH13_28, GH13_29, GH13_3, GH13_30, GH13_31, GH13_32, GH13_33, GH13_34, GH13_35, GH13_36, GH13_37, GH13_38, GH13_39, GH13_4, GH13_40, GH13_41, GH13_42, GH13_5, GH13_6, GH13_7, GH13_8, GH13_9, GH144, GH15, GH17, GH171, GH23, GH3, GH37, GH63, GH73, GH74, GH77, GH78, GH94, GT19, GT20, GT21, GT27, GT28, GT2_Cellulose_synt, GT2_Glyco_tranf_2_2, GT2_Glyco_tranf_2_3, GT2_Glyco_trans_2_3, GT2_Glycos_transf_2, GT30, GT35, GT39, GT4, GT41, GT45, GT5, GT51, GT81, GT83, GT9, PL1, PL12, PL12_1, PL12_2, PL12_3, PL15, PL15_1, PL17, PL1_2, PL1_5, PL22, PL22_1* |
| bin.36 | *AA12, AA3, AA3_1, AA3_2, AA3_3, AA3_4, AA4, AA6, AA7, CBM16, CBM48, CBM50, CE1, CE11, CE12, CE19, CE3, CE4, CE7, CE9, GH102, GH103, GH109, GH119, GH13, GH130, GH13_1, GH13_10, GH13_11, GH13_12, GH13_13, GH13_14, GH13_16, GH13_19, GH13_2, GH13_20, GH13_21, GH13_23, GH13_28, GH13_3, GH13_30, GH13_31, GH13_32, GH13_36, GH13_37, GH13_4, GH13_41, GH13_5, GH13_6, GH13_9, GH15, GH23, GH3, GH33, GH39, GH53, GH74, GH76, GH77, GH78, GH93, GH94, GH99, GT10, GT104, GT105, GT112, GT19, GT20, GT21, GT27, GT28, GT2_Glyco_tranf_2_2, GT2_Glyco_tranf_2_3, GT2_Glyco_tranf_2_4, GT2_Glyco_tranf_2_5, GT2_Glyco_trans_2_3, GT2_Glycos_transf_2, GT30, GT32, GT35, GT39, GT4, GT40, GT41, GT45, GT5, GT51, GT7, GT81, GT83, GT9, GT92, PL1_2, PL22, PL22_1* |
| bin.38 | *AA1, AA12, AA1_1, AA1_2, AA1_3, AA3, AA3_1, AA3_2, AA3_3, AA3_4, AA4, AA6, AA7, CBM50, CE1, CE17, CE19, CE2, CE3, CE7, CE9, GH102, GH103, GH109, GH16, GH16_10, GH16_14, GH16_17, GH16_2, GH16_21, GH16_24, GH16_25, GH16_3, GH16_4, GH16_5, GH16_6, GH16_7, GH16_8, GH16_9, GH171, GH23, GH25, GH3, GH30, GH30_3, GH31, GH39, GH4, GT105, GT19, GT21, GT25, GT26, GT27, GT2_Glyco_tranf_2_2, GT2_Glyco_tranf_2_3, GT2_Glyco_tranf_2_4, GT2_Glyco_trans_2_3, GT2_Glycos_transf_2, GT32, GT39, GT4, GT41, GT45, GT5, GT51, GT81, GT83, GT94, PL1, PL12, PL12_3, PL22, PL22_1, PL31, PL9, PL9_1* |

| | |
|---|---|
| bin.40 | *AA1, AA1_2, AA1_3, AA3, AA3_1, AA3_2, AA3_3, AA3_4, AA4, AA6, AA7, CBM48, CBM50, CE1, CE11, CE12, CE16, CE19, CE2, CE3, CE4, CE7, CE9, GH102, GH103, GH104, GH109, GH120, GH13, GH13_1, GH13_10, GH13_11, GH13_12, GH13_13, GH13_14, GH13_16, GH13_17, GH13_18, GH13_19, GH13_2, GH13_20, GH13_21, GH13_23, GH13_28, GH13_29, GH13_3, GH13_30, GH13_31, GH13_32, GH13_35, GH13_36, GH13_37, GH13_38, GH13_39, GH13_4, GH13_40, GH13_41, GH13_42, GH13_5, GH13_6, GH13_7, GH13_8, GH13_9, GH153, GH23, GH3, GH37, GH65, GH77, GH95, GT1, GT104, GT107, GT113, GT19, GT21, GT27, GT28, GT2_Glyco_tranf_2_2, GT2_Glyco_tranf_2_3, GT2_Glyco_tranf_2_4, GT2_Glyco_trans_2_3, GT2_Glycos_transf_2, GT3, GT30, GT35, GT4, GT41, GT45, GT5, GT51, GT81, GT83, GT9, GT99, PL31, PL41, PL6, PL6_1, PL6_2* |
| bin.41 | *AA12, AA2, AA3_1, AA4, AA5, AA6, AA7, CBM48, CBM50, CE1, CE11, CE12, CE3, CE4, CE7, CE9, GH102, GH103, GH13, GH136, GH13_1, GH13_10, GH13_11, GH13_12, GH13_13, GH13_14, GH13_16, GH13_17, GH13_19, GH13_2, GH13_20, GH13_21, GH13_23, GH13_26, GH13_28, GH13_29, GH13_3, GH13_30, GH13_31, GH13_32, GH13_36, GH13_37, GH13_39, GH13_4, GH13_40, GH13_41, GH13_6, GH13_8, GH13_9, GH23, GH24, GH3, GH33, GH57, GH73, GH74, GH77, GT1, GT10, GT104, GT11, GT19, GT26, GT27, GT28, GT2_Glyco_tranf_2_2, GT2_Glyco_tranf_2_3, GT2_Glyco_tranf_2_4, GT2_Glycos_transf_2, GT30, GT35, GT39, GT4, GT40, GT41, GT5, GT51, GT81, GT83, PL12, PL12_1, PL12_2, PL12_3, PL15, PL15_1, PL15_2, PL17, PL17_1, PL22, PL22_1, PL33, PL33_1, PL35, PL39, PL6, PL6_1, PL6_2* |
| bin.42 | *AA3, AA3_1, AA3_2, AA3_3, AA3_4, AA4, AA6, AA7, CBM50, CE1, CE19, CE3, CE4, CE7, GH103, GH135, GH23, GH24, GH3, GH73, GT104, GT27, GT2_Glyco_tranf_2_2, GT2_Glyco_tranf_2_3, GT2_Glyco_tranf_2_4, GT2_Glyco_trans_2_3, GT2_Glycos_transf_2, GT30, GT4, GT40, GT41, GT5, GT51, GT81, GT83, PL1, PL1_2, PL22, PL22_1* |
| bin.44 | *AA3_1, AA5, AA6, AA7, CBM20, CBM32, CBM4, CBM47, CBM48, CBM50, CBM51, CBM56, CBM62, CBM66, CBM67, CBM9, CE1, CE11, CE12, CE14, CE15, CE19, CE2, CE3, CE4, CE6, CE7, CE9, GH10, GH105, GH106, GH109, GH110, GH117, GH119, GH123, GH125, GH127, GH13, GH130, GH133, GH137, GH13_1, GH13_10, GH13_11, GH13_12, GH13_13, GH13_14, GH13_15, GH13_16, GH13_17, GH13_18, GH13_19, GH13_2, GH13_20, GH13_21, GH13_22, GH13_23, GH13_25, GH13_26, GH13_27, GH13_28, GH13_29, GH13_3, GH13_30, GH13_31, GH13_32, GH13_34, GH13_35, GH13_36, GH13_37, GH13_38, GH13_39, GH13_4, GH13_40, GH13_41, GH13_42, GH13_5, GH13_6, GH13_7, GH13_8, GH13_9, GH142, GH144, GH146, GH148, GH15, GH151, GH154, GH158, GH159, GH16, GH165, GH167, GH16_1, GH16_10, GH16_11, GH16_12, GH16_13, GH16_14, GH16_17, GH16_18, GH16_19, GH16_2, GH16_20, GH16_21, GH16_23, GH16_24, GH16_25, GH16_3, GH16_4,* |

| | |
|---|---|
| | GH16_5, GH16_6, GH16_7, GH16_8, GH16_9, GH171, GH18, GH2, GH20, GH23, GH26, GH28, GH29, GH3, GH30, GH30_1, GH30_2, GH30_3, GH30_4, GH30_5, GH30_6, GH30_7, GH30_8, GH30_9, GH31, GH32, GH33, GH35, GH36, GH37, GH39, GH42, GH43, GH43_1, GH43_10, GH43_11, GH43_12, GH43_13, GH43_14, GH43_15, GH43_16, GH43_17, GH43_18, GH43_19, GH43_2, GH43_22, GH43_23, GH43_24, GH43_26, GH43_27, GH43_28, GH43_29, GH43_3, GH43_30, GH43_31, GH43_32, GH43_33, GH43_34, GH43_35, GH43_36, GH43_37, GH43_4, GH43_5, GH43_6, GH43_7, GH43_8, GH43_9, GH44, GH47, GH5, GH50, GH51, GH53, GH57, GH59, GH5_1, GH5_12, GH5_13, GH5_14, GH5_15, GH5_19, GH5_2, GH5_21, GH5_22, GH5_23, GH5_24, GH5_25, GH5_26, GH5_27, GH5_28, GH5_29, GH5_32, GH5_33, GH5_34, GH5_36, GH5_37, GH5_38, GH5_39, GH5_4, GH5_40, GH5_42, GH5_44, GH5_45, GH5_46, GH5_47, GH5_48, GH5_49, GH5_5, GH5_50, GH5_51, GH5_52, GH5_54, GH5_55, GH5_7, GH5_8, GH5_9, GH63, GH72, GH73, GH74, GH76, GH77, GH78, GH84, GH87, GH88, GH9, GH92, GH93, GH94, GH95, GH97, GH99, GT1, GT113, GT19, GT21, GT27, GT28, GT2_Glyco_tranf_2_2, GT2_Glyco_tranf_2_3, GT2_Glyco_tranf_2_5, GT2_Glyco_trans_2_3, GT2_Glycos_transf_2, GT3, GT30, GT35, GT4, GT40, GT41, GT45, GT5, GT51, GT81, GT83, GT94, PL11, PL11_1, PL12, PL12_1, PL12_3, PL15, PL22, PL22_1, PL33, PL33_1, PL33_2, PL35, PL39, PL4 |

**Table S4.** Host bacteria carrying organic degradation functional genes in sample 2.

| Bin ID | Functional genes |
|---|---|
| bin.2 | *AA1, AA1_2, AA1_3, AA3, AA3_1, AA3_2, AA3_3, AA3_4, AA4, AA7, CBM20, CBM34, CBM50, CBM66, CE1, CE14, CE19, CE3, CE4, CE7, CE9, GH1, GH105, GH109, GH114, GH116, GH117, GH127, GH129, GH13, GH130, GH137, GH139, GH13_1, GH13_10, GH13_11, GH13_12, GH13_13, GH13_14, GH13_15, GH13_16, GH13_17, GH13_18, GH13_19, GH13_2, GH13_20, GH13_21, GH13_22, GH13_23, GH13_24, GH13_26, GH13_27, GH13_28, GH13_29, GH13_3, GH13_30, GH13_31, GH13_32, GH13_33, GH13_34, GH13_35, GH13_36, GH13_37, GH13_38, GH13_39, GH13_4, GH13_40, GH13_41, GH13_42, GH13_5, GH13_6, GH13_7, GH13_8, GH13_9, GH141, GH144, GH146, GH149, GH150, GH158, GH159, GH16, GH160, GH161, GH164, GH165, GH166, GH167, GH168, GH16_1, GH16_10, GH16_11, GH16_13, GH16_14, GH16_17, GH16_18, GH16_19, GH16_2, GH16_20, GH16_21, GH16_24, GH16_25, GH16_27, GH16_3, GH16_4, GH16_5, GH16_6, GH16_7, GH16_8, GH16_9, GH2, GH23, GH26, GH27, GH3, GH31, GH32, GH33, GH35, GH36, GH37, GH4, GH42, GH43, GH43_1, GH43_10, GH43_11, GH43_12, GH43_14, GH43_16, GH43_17, GH43_18, GH43_19, GH43_2, GH43_22, GH43_23, GH43_24, GH43_26, GH43_28, GH43_29, GH43_3, GH43_30, GH43_31, GH43_32, GH43_33, GH43_34, GH43_35, GH43_37, GH43_4, GH43_5, GH43_6, GH43_8, GH43_9, GH5, GH53, GH59, GH5_10, GH5_13, GH5_18, GH5_19, GH5_42, GH5_7, GH65, GH70, GH74, GH78, GH8, GH9, GH93, GH94, GH95, GT1, GT12, GT19, GT27, GT28, GT2_Glyco_tranf_2_2, GT2_Glyco_tranf_2_3, GT2_Glyco_tranf_2_4, GT2_Glyco_trans_2_3, GT2_Glycos_transf_2, GT3, GT33, GT35, GT39, GT4, GT41, GT45, GT5, GT51, GT66, GT81, GT83, GT89, GT94, PL1_12, PL22, PL22_1, PL22_2, PL31, PL41, PL9, PL9_1, PL9_2, PL9_3* |
| bin.5 | *AA1, AA1_1, AA1_2, AA1_3, AA2, AA3, AA3_1, AA3_2, AA4, AA6, AA7, CBM48, CBM50, CE1, CE11, CE16, CE3, CE4, CE5, CE7, CE8, CE9, GH102, GH103, GH113, GH13, GH13_1, GH13_10, GH13_11, GH13_12, GH13_13, GH13_14, GH13_16, GH13_17, GH13_18, GH13_19, GH13_2, GH13_20, GH13_21, GH13_22, GH13_23, GH13_26, GH13_27, GH13_28, GH13_29, GH13_3, GH13_30, GH13_31, GH13_32, GH13_34, GH13_35, GH13_36, GH13_37, GH13_38, GH13_39, GH13_4, GH13_40, GH13_41, GH13_42, GH13_5, GH13_6, GH13_7, GH13_8, GH13_9, GH158, GH17, GH171, GH2, GH23, GH33, GH46, GH5, GH5_1, GH5_14, GH5_15, GH5_22, GH5_23, GH5_25, GH5_26, GH5_29, GH5_32, GH5_33, GH5_36, GH5_37, GH5_38, GH5_39, GH5_44, GH5_45, GH5_46, GH5_47, GH5_48, GH5_49, GH5_5, GH5_50, GH5_52, GH5_54, GH5_7, GH5_8, GH5_9, GH73, GH74, GH93, GT19, GT27, GT28, GT2_Glyco_tranf_2_3, GT2_Glycos_transf_2, GT30, GT35, GT4, GT41, GT45, GT5,* |

| | |
|---|---|
| | *GT51, GT81, GT83, PL15, PL15_1, PL21, PL21_1, PL22, PL31, PL41, PL6, PL9, PL9_1, PL9_2, PL9_4* |
| bin.6 | *AA1, AA12, AA1_1, AA1_2, AA1_3, AA2, AA3, AA3_1, AA3_2, AA3_3, AA3_4, AA4, AA5, AA6, AA7, CBM16, CBM20, CBM23, CBM32, CBM35, CBM37, CBM40, CBM47, CBM48, CBM50, CBM56, CBM6, CBM61, CBM66, CBM67, CBM88, CBM9, CE1, CE11, CE14, CE19, CE2, CE3, CE4, CE7, CE8, CE9, GH1, GH102, GH106, GH109, GH113, GH117, GH120, GH121, GH127, GH13, GH130, GH136, GH137, GH13_1, GH13_10, GH13_11, GH13_12, GH13_13, GH13_14, GH13_15, GH13_16, GH13_17, GH13_18, GH13_19, GH13_2, GH13_20, GH13_21, GH13_22, GH13_23, GH13_26, GH13_27, GH13_28, GH13_29, GH13_3, GH13_30, GH13_31, GH13_32, GH13_34, GH13_35, GH13_36, GH13_37, GH13_38, GH13_39, GH13_4, GH13_40, GH13_41, GH13_42, GH13_5, GH13_6, GH13_7, GH13_8, GH13_9, GH140, GH144, GH146, GH149, GH151, GH158, GH16, GH161, GH163, GH165, GH16_1, GH16_10, GH16_11, GH16_12, GH16_13, GH16_14, GH16_17, GH16_18, GH16_19, GH16_2, GH16_20, GH16_21, GH16_24, GH16_25, GH16_26, GH16_3, GH16_4, GH16_5, GH16_6, GH16_7, GH16_8, GH16_9, GH17, GH171, GH18, GH19, GH2, GH20, GH23, GH25, GH27, GH28, GH29, GH3, GH30, GH30_1, GH30_2, GH30_3, GH30_4, GH30_5, GH30_6, GH30_7, GH30_8, GH30_9, GH31, GH32, GH33, GH36, GH37, GH43, GH43_1, GH43_10, GH43_11, GH43_12, GH43_13, GH43_14, GH43_15, GH43_16, GH43_17, GH43_18, GH43_19, GH43_2, GH43_22, GH43_23, GH43_24, GH43_26, GH43_28, GH43_29, GH43_3, GH43_30, GH43_31, GH43_32, GH43_33, GH43_34, GH43_35, GH43_36, GH43_37, GH43_4, GH43_5, GH43_6, GH43_7, GH43_8, GH43_9, GH5, GH51, GH53, GH57, GH59, GH5_41, GH5_7, GH63, GH65, GH73, GH74, GH76, GH77, GH78, GH87, GH92, GH93, GH95, GH97, GT1, GT105, GT19, GT21, GT22, GT27, GT28, GT2_Glyco_tranf_2_2, GT2_Glyco_tranf_2_3, GT2_Glyco_tranf_2_4, GT2_Glyco_tranf_2_5, GT2_Glyco_trans_2_3, GT2_Glycos_transf_2, GT30, GT35, GT39, GT4, GT40, GT41, GT5, GT51, GT81, GT83, GT9, GT94, PL1, PL11, PL1_2, PL22, PL22_1, PL22_2, PL31, PL41, PL4_1, PL6, PL6_1, PL6_2, PL9, PL9_1, PL9_2, PL9_3, cohesin* |
| bin.7 | *AA12, AA2, AA3, AA3_1, AA3_2, AA3_4, AA4, AA5, AA7, CBM32, CBM48, CE1, CE11, CE3, CE4, CE7, CE9, GH102, GH103, GH108, GH113, GH114, GH13, GH13_1, GH13_10, GH13_11, GH13_12, GH13_13, GH13_14, GH13_16, GH13_17, GH13_19, GH13_2, GH13_20, GH13_21, GH13_23, GH13_28, GH13_29, GH13_3, GH13_30, GH13_31, GH13_32, GH13_36, GH13_37, GH13_41, GH13_42, GH13_5, GH13_6, GH13_8, GH13_9, GH141, GH166, GH23, GH3, GH73, GH74, GH77, GH87, GT1, GT19, GT27, GT28, GT2_Glyco_tranf_2_2, GT2_Glyco_tranf_2_3, GT2_Glyco_tranf_2_4, GT2_Glyco_tranf_2_5, GT2_Glyco_trans_2_3, GT2_Glycos_transf_2, GT33, GT35, GT4, GT41, GT45, GT5, GT51, GT81, GT83, GT87, GT94, PL12, PL12_1, PL12_2, PL12_3, PL14, PL14_3, PL14_4, PL14_5, PL15, PL15_1, PL17, PL39* |

| | |
|---|---|
| bin.9 | *AA2, AA3, AA3_1, AA3_2, AA3_3, AA4, AA7, CBM14, CBM20, CBM50, CBM66, CE1, CE12, CE14, CE19, CE3, CE4, CE7, CE9, GH1, GH109, GH113, GH120, GH13, GH13_1, GH13_10, GH13_11, GH13_12, GH13_13, GH13_14, GH13_15, GH13_16, GH13_17, GH13_18, GH13_19, GH13_2, GH13_20, GH13_21, GH13_22, GH13_23, GH13_24, GH13_26, GH13_27, GH13_28, GH13_29, GH13_3, GH13_30, GH13_31, GH13_32, GH13_34, GH13_35, GH13_36, GH13_37, GH13_38, GH13_39, GH13_4, GH13_40, GH13_41, GH13_42, GH13_5, GH13_6, GH13_7, GH13_8, GH13_9, GH149, GH16, GH166, GH168, GH16_24, GH23, GH26, GH3, GH33, GH37, GH4, GH5, GH5_19, GH5_29, GH5_42, GH5_46, GH5_7, GH63, GH74, GH76, GH77, GH78, GH93, GH94, GT1, GT19, GT20, GT21, GT27, GT28, GT2_Glyco_tranf_2_2, GT2_Glyco_tranf_2_3, GT2_Glycos_transf_2, GT33, GT39, GT4, GT41, GT45, GT47, GT5, GT66, GT81, GT83, GT87, GT94, PL22, PL22_1, PL22_2, PL31, PL41, PL6_2, PL9, PL9_1, PL9_2, PL9_3, PL9_4* |
| bin.11 | *AA1, AA3, AA3_1, AA4, AA5, AA7, CBM20, CBM32, CBM40, CBM47, CBM48, CBM50, CBM9, CE1, CE11, CE14, CE19, CE3, CE4, CE7, CE9, GH10, GH105, GH109, GH119, GH120, GH125, GH13, GH139, GH13_1, GH13_10, GH13_11, GH13_12, GH13_13, GH13_14, GH13_15, GH13_16, GH13_17, GH13_18, GH13_19, GH13_2, GH13_20, GH13_21, GH13_22, GH13_23, GH13_24, GH13_26, GH13_27, GH13_28, GH13_29, GH13_3, GH13_30, GH13_31, GH13_32, GH13_34, GH13_35, GH13_36, GH13_37, GH13_38, GH13_39, GH13_4, GH13_40, GH13_41, GH13_42, GH13_5, GH13_6, GH13_7, GH13_8, GH13_9, GH144, GH151, GH158, GH16, GH163, GH16_1, GH16_10, GH16_13, GH16_14, GH16_17, GH16_18, GH16_19, GH16_2, GH16_21, GH16_24, GH16_25, GH16_3, GH16_4, GH16_5, GH16_6, GH16_7, GH16_8, GH16_9, GH171, GH19, GH2, GH20, GH23, GH25, GH27, GH29, GH3, GH33, GH35, GH36, GH39, GH42, GH43, GH43_1, GH43_10, GH43_11, GH43_12, GH43_16, GH43_2, GH43_28, GH43_29, GH43_3, GH43_30, GH43_31, GH43_32, GH43_33, GH43_34, GH43_4, GH43_5, GH5, GH51, GH53, GH55, GH63, GH70, GH73, GH74, GH76, GH77, GH78, GH87, GH92, GH93, GH95, GH97, GT11, GT12, GT13, GT19, GT21, GT27, GT28, GT2_Glyco_tranf_2_2, GT2_Glyco_tranf_2_3, GT2_Glyco_tranf_2_4, GT2_Glyco_tranf_2_5, GT2_Glyco_trans_2_3, GT2_Glycos_transf_2, GT3, GT30, GT35, GT4, GT40, GT41, GT45, GT5, GT51, GT74, GT81, GT83, GT9, GT94, PL11_2, PL22, PL22_1, PL22_2, PL31, PL32, PL41, PL9_2* |
| bin.14 | *AA1, AA1_1, AA1_2, AA1_3, AA3, AA3_1, AA3_2, AA4, AA7, CBM32, CBM34, CBM50, CBM66, CBM9, CE1, CE11, CE16, CE19, CE4, CE7, CE9, GH1, GH100, GH103, GH109, GH116, GH127, GH13, GH133, GH13_1, GH13_10, GH13_11, GH13_12, GH13_13, GH13_14, GH13_16, GH13_17, GH13_18, GH13_19, GH13_2, GH13_20, GH13_21, GH13_22, GH13_23, GH13_25, GH13_26, GH13_27, GH13_28, GH13_29, GH13_3, GH13_30, GH13_31, GH13_32, GH13_34, GH13_35, GH13_36, GH13_37,* |

| | |
|---|---|
| | *GH13_38, GH13_39, GH13_4, GH13_40, GH13_41, GH13_42, GH13_5, GH13_6, GH13_7, GH13_9, GH146, GH15, GH171, GH20, GH23, GH26, GH31, GH38, GH39, GH42, GH5, GH57, GH5_1, GH5_12, GH5_2, GH5_22, GH5_25, GH5_26, GH5_36, GH5_37, GH5_38, GH5_39, GH5_4, GH5_46, GH5_47, GH5_48, GH5_5, GH5_52, GH5_54, GH5_8, GH5_9, GH65, GH76, GH78, GT1, GT11, GT112, GT19, GT21, GT23, GT26, GT27, GT28, GT2_Glyco_tranf_2_2, GT2_Glyco_tranf_2_3, GT2_Glyco_tranf_2_4, GT2_Glyco_tranf_2_5, GT2_Glyco_trans_2_3, GT2_Glycos_transf_2, GT3, GT30, GT35, GT39, GT4, GT40, GT41, GT45, GT5, GT51, GT81, GT83, GT9, GT94, PL12, PL22, PL22_1, PL22_2, PL27, PL31, PL33, PL33_1, PL33_2, PL35, PL39, PL9, PL9_1, PL9_2* |
| bin.15 | *AA1, AA1_2, AA1_3, AA4, CBM50, CE4, GH103, GH116, GH142, GH15, GH18, GH23, GH37, GH38, GH57, GH63, GH74, GH78, GH94, GT1, GT21, GT27, GT28, GT2_Chitin_synth_2, GT2_Glyco_tranf_2_2, GT2_Glyco_tranf_2_3, GT2_Glyco_trans_2_3, GT2_Glycos_transf_2, GT3, GT35, GT4, GT41, GT45, GT5, GT51, GT81, GT83, cohesin, dockerin* |
| bin.16 | *AA1, AA1_1, AA1_2, AA1_3, AA3, AA3_1, AA3_2, AA3_4, AA5, AA6, CBM40, CBM48, CBM50, CBM56, CBM68, CE1, CE12, CE14, CE19, CE2, CE3, CE4, CE7, CE9, GH1, GH109, GH119, GH13, GH13_1, GH13_10, GH13_11, GH13_12, GH13_13, GH13_14, GH13_15, GH13_16, GH13_17, GH13_18, GH13_19, GH13_2, GH13_20, GH13_21, GH13_22, GH13_23, GH13_24, GH13_26, GH13_27, GH13_28, GH13_29, GH13_3, GH13_30, GH13_31, GH13_32, GH13_34, GH13_35, GH13_36, GH13_37, GH13_38, GH13_39, GH13_4, GH13_40, GH13_41, GH13_42, GH13_5, GH13_6, GH13_7, GH13_8, GH13_9, GH144, GH170, GH18, GH2, GH23, GH31, GH5, GH5_5, GH73, GH74, GH99, GT1, GT19, GT21, GT26, GT27, GT28, GT2_Chitin_synth_2, GT2_Glyco_tranf_2_2, GT2_Glyco_tranf_2_3, GT2_Glyco_tranf_2_4, GT2_Glyco_trans_2_3, GT2_Glycos_transf_2, GT3, GT35, GT4, GT41, GT45, GT5, GT51, GT66, GT81, GT83, PL10, PL10_1, PL22, PL31, PL41, SLH* |
| bin.17 | *AA12, AA2, AA3, AA3_1, AA3_2, AA4, AA7, CBM48, CBM50, CE1, CE19, CE3, CE5, CE7, CE9, GH103, GH109, GH13, GH13_1, GH13_10, GH13_11, GH13_12, GH13_13, GH13_14, GH13_16, GH13_17, GH13_19, GH13_2, GH13_20, GH13_21, GH13_23, GH13_27, GH13_28, GH13_29, GH13_3, GH13_30, GH13_31, GH13_32, GH13_35, GH13_36, GH13_37, GH13_38, GH13_39, GH13_4, GH13_40, GH13_41, GH13_42, GH13_5, GH13_6, GH13_8, GH13_9, GH15, GH171, GH23, GH3, GH33, GH37, GH39, GH74, GH77, GH78, GH79, GH93, GT1, GT12, GT19, GT20, GT27, GT28, GT2_Glyco_tranf_2_2, GT2_Glyco_tranf_2_3, GT2_Glycos_transf_2, GT35, GT39, GT4, GT41, GT5, GT51, GT7, GT81, GT83, PL22, PL22_1* |
| bin.21 | *AA1, AA1_1, AA1_2, AA1_3, AA3, AA3_1, AA3_2, CBM50, CE1, CE19, CE3, CE7, CE8, CE9, GH1, GH10, GH103, GH117, GH119, GH130, GH137, GH158, GH23, GH32, GH33, GH4, GH42, GH43, GH43_26, GH43_34, GH5, GH5_47, GH74, GH93,* |

| | |
|---|---|
| | *GT21, GT26, GT27, GT2_Chitin_synth_2, GT2_Glyco_tranf_2_2, GT2_Glyco_tranf_2_3, GT2_Glyco_tranf_2_4, GT2_Glyco_tranf_2_5, GT2_Glyco_trans_2_3, GT2_Glycos_transf_2, GT3, GT39, GT4, GT40, GT41, GT45, GT5, GT51, GT81, GT83, GT94, PL31, PL41, PL9, PL9_1, PL9_2, PL9_3* |
| bin.22 | *AA2, AA3_1, CBM20, CBM32, CBM47, CBM48, CBM50, CBM67, CE1, CE11, CE14, CE19, CE4, CE6, CE7, CE9, GH109, GH119, GH13, GH13_1, GH13_10, GH13_11, GH13_12, GH13_13, GH13_14, GH13_15, GH13_16, GH13_17, GH13_19, GH13_2, GH13_20, GH13_21, GH13_22, GH13_23, GH13_24, GH13_26, GH13_27, GH13_28, GH13_29, GH13_3, GH13_30, GH13_31, GH13_32, GH13_34, GH13_35, GH13_36, GH13_37, GH13_38, GH13_39, GH13_4, GH13_40, GH13_41, GH13_42, GH13_5, GH13_6, GH13_7, GH13_8, GH13_9, GH158, GH16, GH16_1, GH16_10, GH16_13, GH16_2, GH16_21, GH16_24, GH16_3, GH16_4, GH16_5, GH16_6, GH16_7, GH16_8, GH16_9, GH171, GH2, GH25, GH3, GH30, GH30_1, GH30_2, GH30_3, GH30_4, GH30_6, GH30_7, GH30_8, GH30_9, GH31, GH43, GH43_14, GH43_18, GH43_2, GH43_28, GH43_29, GH43_3, GH43_31, GH43_33, GH43_34, GH43_4, GH43_8, GH53, GH63, GH70, GH73, GH77, GH87, GH97, GT113, GT19, GT21, GT27, GT28, GT2_Glyco_tranf_2_2, GT2_Glyco_tranf_2_3, GT2_Glyco_tranf_2_4, GT2_Glyco_tranf_2_5, GT2_Glyco_trans_2_3, GT2_Glycos_transf_2, GT33, GT35, GT4, GT41, GT45, GT5, GT51, GT74, GT81, GT83, PL22, PL22_1* |
| bin.23 | *AA3, AA3_1, AA3_2, AA4, AA5, AA6, AA7, CBM50, CE1, CE11, CE19, CE7, CE9, GH103, GH109, GH19, GH23, GH24, GH3, GT1, GT113, GT19, GT21, GT27, GT28, GT2_Glyco_tranf_2_2, GT2_Glyco_tranf_2_3, GT2_Glyco_tranf_2_4, GT2_Glycos_transf_2, GT30, GT4, GT41, GT45, GT5, PL22, PL22_2* |
| bin.25 | *AA3, AA3_1, AA3_2, AA4, AA7, CBM50, CBM67, CE9, GH23, GH33, GH74, GH93, GT1, GT21, GT22, GT26, GT27, GT28, GT2_Glyco_tranf_2_2, GT2_Glyco_tranf_2_3, GT2_Glyco_tranf_2_4, GT2_Glyco_trans_2_3, GT2_Glycos_transf_2, GT39, GT4, GT40, GT41, GT45, GT5, GT51, GT81, GT83, GT94* |
| bin.26 | *AA1, AA10, AA1_1, AA1_2, AA1_3, AA3_1, AA7, CBM12, CBM34, CBM5, CBM50, CBM67, CBM73, CE1, CE11, CE16, CE9, GH1, GH103, GH109, GH13, GH138, GH13_1, GH13_10, GH13_11, GH13_12, GH13_13, GH13_14, GH13_15, GH13_16, GH13_17, GH13_18, GH13_19, GH13_2, GH13_20, GH13_21, GH13_22, GH13_23, GH13_24, GH13_26, GH13_27, GH13_28, GH13_29, GH13_3, GH13_30, GH13_31, GH13_32, GH13_33, GH13_34, GH13_35, GH13_36, GH13_37, GH13_38, GH13_39, GH13_4, GH13_40, GH13_41, GH13_42, GH13_5, GH13_6, GH13_7, GH13_8, GH13_9, GH158, GH18, GH2, GH20, GH23, GH5, GH70, GT1, GT19, GT21, GT27, GT2_Cellulose_synt, GT2_Glyco_tranf_2_2, GT2_Glyco_tranf_2_3, GT2_Glyco_trans_2_3, GT2_Glycos_transf_2, GT4, GT41, GT45, GT5, GT51, GT76, GT81, GT9* |

| | |
|---|---|
| bin.32 | *AA1, AA1_1, AA1_2, AA1_3, AA3, AA3_1, AA3_2, AA4, AA6, AA7, CBM16, CBM34, CBM4, CBM41, CBM48, CBM50, CBM83, CBM85, CE1, CE14, CE4, CE7, CE9, GH1, GH103, GH109, GH114, GH117, GH13, GH130, GH137, GH13_1, GH13_10, GH13_11, GH13_12, GH13_13, GH13_14, GH13_15, GH13_16, GH13_17, GH13_18, GH13_19, GH13_2, GH13_20, GH13_21, GH13_22, GH13_23, GH13_24, GH13_26, GH13_27, GH13_28, GH13_29, GH13_3, GH13_30, GH13_31, GH13_32, GH13_34, GH13_35, GH13_36, GH13_37, GH13_38, GH13_39, GH13_4, GH13_40, GH13_41, GH13_42, GH13_5, GH13_6, GH13_7, GH13_8, GH13_9, GH159, GH164, GH166, GH167, GH168, GH170, GH171, GH18, GH2, GH23, GH28, GH3, GH31, GH32, GH35, GH36, GH4, GH42, GH43, GH43_1, GH43_10, GH43_11, GH43_12, GH43_13, GH43_14, GH43_15, GH43_16, GH43_17, GH43_18, GH43_19, GH43_2, GH43_22, GH43_23, GH43_24, GH43_26, GH43_27, GH43_28, GH43_29, GH43_3, GH43_30, GH43_31, GH43_32, GH43_33, GH43_34, GH43_35, GH43_36, GH43_37, GH43_4, GH43_5, GH43_6, GH43_7, GH43_8, GH43_9, GH5, GH50, GH51, GH55, GH57, GH5_36, GH65, GH66, GH70, GH73, GH74, GH94, GH95, GT1, GT113, GT12, GT19, GT21, GT27, GT28, GT2_Chitin_synth_2, GT2_Glyco_tranf_2_2, GT2_Glyco_tranf_2_3, GT2_Glyco_tranf_2_4, GT2_Glyco_trans_2_3, GT2_Glycos_transf_2, GT3, GT35, GT4, GT41, GT45, GT5, GT51, GT81, PL1, PL12, PL12_1, PL12_2, PL12_3, PL17, PL40* |
| bin.33 | *AA3, AA3_1, AA3_2, AA3_3, AA3_4, AA4, AA5, AA6, AA7, CBM50, CE1, CE4, CE7, CE9, GH103, GH19, GH23, GH3, GH33, GH74, GT1, GT21, GT27, GT28, GT2_Glyco_tranf_2_2, GT2_Glyco_tranf_2_3, GT2_Glyco_tranf_2_4, GT2_Glycos_transf_2, GT33, GT39, GT4, GT41, GT5, GT51, GT81, GT83* |
| bin.34 | *AA3, AA3_1, AA3_2, AA4, AA7, CBM50, CE1, CE11, CE14, CE7, CE9, GH102, GH23, GH3, GT104, GT19, GT21, GT27, GT28, GT2_Cellulose_synt, GT2_Glyco_tranf_2_2, GT2_Glyco_tranf_2_3, GT2_Glyco_trans_2_3, GT2_Glycos_transf_2, GT30, GT41, GT45, GT51, GT81, GT83* |
| bin.36 | *AA12, AA4, AA7, CBM50, CE9, GH1, GH113, GH128, GH158, GH2, GH23, GH35, GH42, GH5, GH5_10, GH5_19, GH5_29, GH5_4, GH5_5, GH74, GT1, GT20, GT21, GT26, GT27, GT28, GT2_Glyco_tranf_2_2, GT2_Glyco_tranf_2_3, GT2_Glyco_tranf_2_4, GT2_Glyco_trans_2_3, GT2_Glycos_transf_2, GT3, GT39, GT4, GT40, GT41, GT45, GT5, GT51, GT81, GT83, GT94, cohesin* |
| bin.37 | *AA3, AA3_1, AA3_2, AA3_3, AA4, AA7, CBM16, CBM2, CBM22, CBM34, CBM35, CBM38, CBM50, CBM66, CBM67, CBM70, CBM84, CBM9, CE1, CE11, CE12, CE14, CE19, CE2, CE4, CE6, CE7, CE9, GH10, GH100, GH103, GH105, GH106, GH109, GH127, GH13, GH130, GH133, GH13_1, GH13_10, GH13_11, GH13_12, GH13_13, GH13_14, GH13_16, GH13_17, GH13_18,* |

| | |
|---|---|
| | *GH13_19, GH13_2, GH13_20, GH13_21, GH13_22, GH13_23, GH13_25, GH13_26, GH13_27, GH13_28, GH13_29, GH13_3, GH13_30, GH13_31, GH13_32, GH13_34, GH13_35, GH13_36, GH13_37, GH13_38, GH13_39, GH13_4, GH13_40, GH13_41, GH13_42, GH13_5, GH13_6, GH13_7, GH13_9, GH141, GH146, GH15, GH151, GH2, GH20, GH23, GH26, GH29, GH31, GH33, GH38, GH39, GH4, GH42, GH5, GH51, GH57, GH65, GH74, GH76, GH78, GH93, GH95, GT1, GT105, GT12, GT19, GT21, GT26, GT27, GT28, GT2_Glyco_tranf_2_2, GT2_Glyco_tranf_2_3, GT2_Glyco_tranf_2_4, GT2_Glyco_tranf_2_5, GT2_Glyco_trans_2_3, GT2_Glycos_transf_2, GT3, GT30, GT35, GT39, GT4, GT41, GT45, GT5, GT51, GT81, GT83, GT9, PL1, PL22, PL22_1, PL22_2, PL27, PL31, PL35, PL41, PL6, PL6_1, PL6_2, PL9, PL9_1, PL9_2, PL9_3* |
| bin.38 | *AA1, AA1_2, AA1_3, AA3_1, AA5, AA7, CBM32, CBM50, CE1, CE11, CE14, CE19, CE4, CE7, CE9, GH109, GH119, GH138, GH14, GH151, GH158, GH171, GH2, GH20, GH25, GH29, GH3, GH33, GH35, GH42, GH73, GH74, GH93, GT113, GT12, GT19, GT21, GT27, GT28, GT2_Glyco_tranf_2_2, GT2_Glyco_tranf_2_3, GT2_Glyco_tranf_2_4, GT2_Glyco_trans_2_3, GT2_Glycos_transf_2, GT30, GT33, GT4, GT40, GT41, GT45, GT5, GT51, GT61, GT81, GT87, GT9, PL22, PL22_1, PL31, PL32, PL4, PL41, PL6, PL6_1, PL6_2, PL9, PL9_2* |
| bin.40 | *AA12, AA2, AA3, AA3_1, AA3_2, AA4, AA7, CBM16, CBM50, CBM61, CBM70, CBM86, CBM9, CE1, CE11, CE12, CE19, CE2, CE3, CE4, CE7, CE9, GH100, GH105, GH109, GH13, GH133, GH136, GH13_1, GH13_17, GH13_2, GH13_20, GH13_21, GH13_23, GH13_25, GH13_29, GH13_31, GH13_36, GH13_38, GH15, GH171, GH23, GH3, GH32, GH33, GH37, GH43, GH43_1, GH43_10, GH43_11, GH43_12, GH43_13, GH43_14, GH43_15, GH43_16, GH43_17, GH43_18, GH43_19, GH43_2, GH43_22, GH43_24, GH43_26, GH43_28, GH43_29, GH43_3, GH43_30, GH43_31, GH43_32, GH43_33, GH43_34, GH43_36, GH43_37, GH43_4, GH43_5, GH43_8, GH43_9, GH55, GH74, GH76, GH78, GH93, GH94, GT1, GT112, GT19, GT21, GT27, GT28, GT2_Glyco_tranf_2_2, GT2_Glyco_tranf_2_3, GT2_Glyco_trans_2_3, GT2_Glycos_transf_2, GT33, GT39, GT4, GT41, GT45, GT5, GT51, GT81, GT83, GT9, PL11_2, PL12, PL12_1, PL12_2, PL12_3, PL15, PL15_1, PL17, PL17_1, PL22, PL22_1, PL22_2, PL39, PL40, PL9_4* |
| bin.41 | *AA1, AA1_1, AA1_2, AA1_3, AA3, AA3_1, AA3_2, AA3_3, AA3_4, AA4, AA5, AA5_2, AA6, AA7, CBM16, CBM34, CBM35, CBM48, CBM50, CBM66, CE1, CE14, CE19, CE4, CE7, CE9, GH1, GH10, GH109, GH117, GH128, GH13, GH130, GH136, GH137, GH13_1, GH13_10, GH13_11, GH13_12, GH13_13, GH13_14, GH13_15, GH13_16, GH13_17, GH13_18, GH13_19, GH13_2, GH13_20, GH13_21, GH13_22, GH13_23, GH13_24, GH13_26, GH13_27, GH13_28, GH13_29, GH13_3, GH13_30, GH13_31, GH13_32, GH13_34, GH13_35, GH13_36, GH13_37, GH13_38, GH13_39, GH13_4, GH13_40, GH13_41, GH13_42, GH13_5,* |

| | |
|---|---|
| | *GH13_6, GH13_7, GH13_8, GH13_9, GH142, GH144, GH150, GH159, GH165, GH18, GH23, GH3, GH32, GH33, GH35, GH37, GH39, GH4, GH42, GH43, GH43_18, GH43_23, GH43_3, GH43_30, GH43_32, GH43_33, GH43_34, GH5, GH51, GH55, GH5_47, GH63, GH74, GH78, GH79, GH93, GH94, GH99, GT1, GT12, GT27, GT28, GT2_Glyco_tranf_2_2, GT2_Glyco_tranf_2_3, GT2_Glyco_trans_2_3, GT2_Glycos_transf_2, GT3, GT35, GT39, GT4, GT40, GT41, GT45, GT5, GT51, GT76, GT81, GT83, PL11, PL11_1, PL22, PL22_1, PL9, PL9_1, PL9_2* |
| bin.44 | *AA12, AA3_1, AA4, AA6, AA7, CBM50, CBM56, CE1, CE9, GH1, GH10, GH158, GH33, GH35, GH42, GH5, GH5_19, GH5_28, GH5_29, GH5_4, GH5_47, GH5_5, GH74, GT1, GT20, GT21, GT27, GT28, GT2_Glyco_tranf_2_2, GT2_Glyco_tranf_2_3, GT2_Glyco_tranf_2_4, GT2_Glyco_trans_2_3, GT2_Glycos_transf_2, GT3, GT39, GT4, GT41, GT5, GT51, GT81, GT83, PL22, cohesin* |
| bin.45 | *AA3, AA3_1, AA3_2, AA3_3, AA3_4, AA4, AA5, AA5_2, AA6, AA7, CBM50, CE1, CE12, CE3, CE9, GH102, GH103, GH114, GH119, GH166, GH167, GH23, GH24, GH3, GH42, GH73, GT27, GT2_Glyco_tranf_2_3, GT2_Glyco_trans_2_3, GT2_Glycos_transf_2, GT4, GT41, GT5, GT51, GT81, GT83, PL1, PL1_2, PL22* |
| bin.46 | *AA3, AA3_1, AA3_2, AA4, AA6, AA7, CBM48, CBM50, CBM56, CE1, CE4, CE7, CE9, GH101, GH109, GH113, GH13, GH13_1, GH13_10, GH13_11, GH13_12, GH13_13, GH13_14, GH13_15, GH13_16, GH13_17, GH13_18, GH13_19, GH13_2, GH13_20, GH13_21, GH13_22, GH13_23, GH13_24, GH13_26, GH13_27, GH13_28, GH13_29, GH13_3, GH13_30, GH13_31, GH13_32, GH13_34, GH13_35, GH13_36, GH13_37, GH13_38, GH13_39, GH13_4, GH13_40, GH13_41, GH13_42, GH13_5, GH13_6, GH13_7, GH13_8, GH13_9, GH170, GH18, GH32, GH4, GH73, GH74, GT1, GT12, GT13, GT19, GT21, GT27, GT28, GT2_Chitin_synth_2, GT2_Glyco_tranf_2_2, GT2_Glyco_tranf_2_3, GT2_Glyco_tranf_2_4, GT2_Glyco_trans_2_3, GT2_Glycos_transf_2, GT35, GT39, GT4, GT41, GT5, GT51, GT66, GT81, SLH* |
| bin.48 | *AA12, AA3, AA3_1, AA3_2, AA7, CBM40, CBM50, CBM51, CE1, CE14, CE19, CE7, GH109, GH18, GH3, GH39, GH5, GH5_2, GH5_22, GH5_26, GH5_39, GH5_46, GH5_47, GH5_5, GH5_52, GH5_8, GH73, GT1, GT105, GT11, GT113, GT20, GT21, GT22, GT26, GT27, GT28, GT2_Glyco_tranf_2_2, GT2_Glyco_tranf_2_3, GT2_Glyco_tranf_2_4, GT2_Glyco_trans_2_3, GT2_Glycos_transf_2, GT3, GT39, GT4, GT40, GT41, GT45, GT5, GT51, GT66, GT70, GT81, GT83, GT87, GT94, cohesin* |

**Table S5.** Host bacteria carrying organic degradation functional genes in sample 3.

| Bin ID | Functional genes |
|---|---|
| bin. 4 | *CBM16, CBM32, CBM4, CBM50, CBM9, CE11, CE14, CE1, CE2, CE3, CE4, CE7, CE8, CE9, GH100, GH105, GH119, GH13, GH15, GH23, GH28, GH33, GH3, GH44, GH57, GH5, GH70, GH74, GH76, GH77, GH78, GH92, GH93, GH94, GH9, GT1, GT21, GT27, GT28, GT30, GT35, GT39, GT41, GT45, GT4, GT51, GT5, GT81, GT83, GT9, PL10, PL1, PL22, GT94, AA3, AA4, AA5, AA6, AA7, CBM66, AA12, CBM70, GH133, GH144, GT2_Glycos_transf_2, GT2_Glyco_tranf_2_2, GT2_Glyco_tranf_2_3, GT2_Glyco_trans_2_3, AA3_1, GH13_10, GH13_11, GH13_12, GH13_13, GH13_14, GH13_15, GH13_16, GH13_17, GH13_18, GH13_19, GH13_1, GH13_20, GH13_21, GH13_22, GH13_23, GH13_25, GH13_26, GH13_27, GH13_28, GH13_29, GH13_2, GH13_30, GH13_31, GH13_32, GH13_34, GH13_35, GH13_36, GH13_37, GH13_38, GH13_39, GH13_3, GH13_40, GH13_41, GH13_42, GH13_4, GH13_5, GH13_6, GH13_7, GH13_8, GH13_9, GH5_10, GH5_19, GH5_1, GH5_25, GH5_30, GH5_41, GH5_42, GH5_46, GH5_7, PL10_1, PL10_2, PL10_3, PL11_2, PL22_1, PL22_2, GH109, GT105, CE19, GH171, GT112, PL41* |
| bin. 5 | *CBM50, CE11, CE12, CE16, CE1, CE2, CE3, CE4, CE7, CE9, GH102, GH103, GH113, GH13, GH17, GH19, GH23, GH33, GH3, GH5, GH73, GH74, GH93, GT13, GT19, GT20, GT21, GT26, GT27, GT28, GT30, GT39, GT41, GT45, GT4, GT51, GT5, GT81, GT83, GT9, PL22, AA2, AA3, AA4, AA6, AA7, GT104, GT2_Glycos_transf_2, GT2_Glyco_tranf_2_2, GT2_Glyco_tranf_2_3, GT2_Glyco_trans_2_3, AA1_1, AA1_2, AA1_3, AA3_1, AA3_2, AA3_4, GH13_10, GH13_11, GH13_12, GH13_13, GH13_14, GH13_16, GH13_17, GH13_18, GH13_19, GH13_1, GH13_20, GH13_21, GH13_22, GH13_23, GH13_26, GH13_27, GH13_28, GH13_29, GH13_2, GH13_30, GH13_31, GH13_32, GH13_33, GH13_34, GH13_35, GH13_36, GH13_37, GH13_38, GH13_39, GH13_3, GH13_40, GH13_41, GH13_42, GH13_4, GH13_5, GH13_6, GH13_7, GH13_8, GH13_9, GH5_14, GH5_15, GH5_1, GH5_22, GH5_23, GH5_25, GH5_27, GH5_28, GH5_29, GH5_33, GH5_36, GH5_37, GH5_38, GH5_39, GH5_40, GH5_44, GH5_45, GH5_46, GH5_47, GH5_49, GH5_4, GH5_50, GH5_52, GH5_54, GH5_5, GH5_8, GH5_9, PL22_1, PL22_2, PL6_1, AA1, GH109, GH171* |
| bin. 6 | *CBM48, CBM50, CE11, CE1, CE3, CE4, CE9, GH102, GH103, GH105, GH13, GH15, GH19, GH23, GH33, GH38, GH3, GH57, GH78, GH93, GT19, GT1, GT20, GT27, GT28, GT30, GT35, GT39, GT41, GT4, GT51, GT5, GT81, GT83, GT9, PL22, PL6, PL9, AA4, AA5, AA6, AA7, AA12, PL31, GT2_Glycos_transf_2, GT2_Glyco_tranf_2_3, AA3_2, AA5_1, AA5_2, GH13_10, GH13_11, GH13_12, GH13_13, GH13_14, GH13_16, GH13_17, GH13_19, GH13_1, GH13_20, GH13_21, GH13_23, GH13_26, GH13_28,* |

| | |
|---|---|
| | GH13_29, GH13_2, GH13_30, GH13_31, GH13_32, GH13_36, GH13_37, GH13_3, GH13_40, GH13_41, GH13_4, GH13_5, GH13_8, GH13_9, PL22_1, PL6_1, GH171, PL41 |
| bin. 8 | CBM13, CBM16, CBM22, CBM50, CBM61, CE11, CE12, CE16, CE1, CE3, CE4, CE7, CE9, GH102, GH114, GH23, GH24, GH33, GH35, GH3, GH42, GH73, GH74, GT19, GT21, GT25, GT27, GT28, GT30, GT41, GT4, GT51, GT5, GT7, GT81, GT83, PL22, AA3, AA4, AA6, AA7, GH135, GT104, GT2_Glycos_transf_2, GT2_Glyco_tranf_2_2, GT2_Glyco_tranf_2_3, GT2_Glyco_tranf_2_4, GT2_Glyco_trans_2_3, AA1_1, AA1_2, AA1_3, AA3_1, AA3_2, AA3_3, AA3_4, PL22_1, GH166, GH167, AA1 |
| bin. 9 | CBM16, CBM20, CBM22, CBM32, CBM34, CBM37, CBM40, CBM47, CBM48, CBM4, CBM50, CBM61, CBM6, CBM9, CE11, CE12, CE14, CE1, CE3, CE4, CE7, CE8, CE9, GH103, GH105, GH110, GH116, GH117, GH119, GH120, GH121, GH13, GH1, GH23, GH25, GH2, GH30, GH31, GH32, GH33, GH35, GH36, GH37, GH38, GH39, GH3, GH42, GH43, GH44, GH46, GH4, GH51, GH53, GH57, GH5, GH63, GH70, GH74, GH77, GH78, GH79, GH87, GH93, GH94, GT14, GT21, GT27, GT35, GT39, GT40, GT41, GT45, GT4, GT51, GT5, GT66, GT76, GT81, GT83, GT87, PL11, PL14, PL1, PL22, PL6, PL9, SLH, GH127, GH128, GH129, GH130, GT94, AA3, AA4, AA5, AA7, CBM66, GH136, GH142, GH144, PL42, GH150, GH158, GH164, GH165, PL31, GT2_Glycos_transf_2, GT2_Glyco_tranf_2_2, GT2_Glyco_tranf_2_3, GT2_Glyco_tranf_2_4, GT2_Glyco_trans_2_3, AA1_1, AA1_2, AA1_3, AA3_1, AA3_2, AA3_3, AA5_1, AA5_2, GH13_10, GH13_11, GH13_12, GH13_13, GH13_14, GH13_15, GH13_16, GH13_17, GH13_18, GH13_19, GH13_1, GH13_20, GH13_21, GH13_22, GH13_23, GH13_26, GH13_27, GH13_28, GH13_29, GH13_2, GH13_30, GH13_31, GH13_32, GH13_34, GH13_35, GH13_36, GH13_37, GH13_38, GH13_39, GH13_3, GH13_40, GH13_41, GH13_42, GH13_4, GH13_5, GH13_6, GH13_7, GH13_8, GH13_9, GH30_3, GH5_42, PL11_1, PL1_2, PL22_1, PL22_2, PL6_1, PL6_2, PL9_1, PL9_2, PL9_3, PL9_4, CBM35, GH167, AA1, CBM84, PL41 |
| bin. 10 | CBM50, CE14, CE1, CE3, CE7, GH116, GH15, GH18, GH1, GH37, GH39, GH42, GH51, GH57, GH5, GH73, GH78, GT12, GT1, GT20, GT21, GT26, GT27, GT28, GT35, GT39, GT3, GT40, GT41, GT45, GT4, GT51, GT5, GT66, GT74, GT76, GT81, GT83, GT87, GT94, AA7, AA12, GH142, GT2_Glycos_transf_2, GT2_Glyco_tranf_2_2, GT2_Glyco_tranf_2_3, GT2_Glyco_tranf_2_4, GT2_Glyco_tranf_2_5, GT2_Glyco_trans_2_3, GH5_10, GH5_19, GH5_27, GH5_29, GH5_4, GT105, CE19 |
| bin. 11 | CBM48, CBM50, CBM8, CE11, CE12, CE1, CE2, CE3, CE4, CE7, CE8, CE9, cohesin, GH100, GH105, GH116, GH13, GH15, GH20, GH23, GH33, GH37, GH39, GH3, GH42, GH5, GH63, GH74, GH76, GH77, GH78, GH93, GH94, GT12, GT19, GT20, GT21, GT27, GT28, GT30, GT35, GT39, GT3, GT40, GT41, GT45, GT4, GT51, GT5, GT70, GT81, GT83, GT9, PL10, PL12, PL14, |

| | |
|---|---|
| | *PL1, PL22, PL4, PL6, PL9, GT94, AA3, AA4, AA6, AA7, CBM66, GH133, GH142, GT2_Glycos_transf_2, GT2_Glyco_tranf_2_2, GT2_Glyco_tranf_2_3, GT2_Glyco_tranf_2_4, GT2_Glyco_tranf_2_5, GT2_Glyco_trans_2_3, AA1_1, AA1_2, AA1_3, AA3_1, GH13_10, GH13_11, GH13_12, GH13_13, GH13_14, GH13_16, GH13_17, GH13_19, GH13_1, GH13_20, GH13_21, GH13_23, GH13_25, GH13_26, GH13_27, GH13_28, GH13_29, GH13_2, GH13_30, GH13_31, GH13_32, GH13_34, GH13_35, GH13_36, GH13_37, GH13_38, GH13_39, GH13_3, GH13_40, GH13_41, GH13_42, GH13_4, GH13_5, GH13_6, GH13_7, GH13_8, GH13_9, GH5_13, PL10_1, PL12_1, PL12_2, PL12_3, PL1_2, PL14_3, PL22_1, PL22_2, PL6_1, PL9_1, PL9_2, PL9_3, AA1, GH109, PL41* |
| bin. 13 | *CBM13, CBM50, CE11, CE12, CE1, CE2, CE3, CE4, CE7, CE9, GH103, GH18, GH23, GH25, GH3, GT12, GT19, GT1, GT21, GT25, GT27, GT28, GT30, GT32, GT33, GT41, GT45, GT4, GT51, GT5, PL12, PL15, PL17, AA3, AA6, AA12, GH153, GT2_Glycos_transf_2, GT2_Glyco_tranf_2_2, GT2_Glyco_tranf_2_3, GT2_Glyco_tranf_2_4, AA3_1, PL12_1, PL12_2, PL12_3, CE17, GH109, GT113* |
| bin. 15 | *CBM16, CBM20, CBM32, CBM34, CBM40, CBM48, CBM50, CBM53, CBM56, CBM9, CE11, CE12, CE14, CE1, CE3, CE4, CE7, CE9, GH113, GH13, GH20, GH23, GH2, GH33, GH3, GH57, GH5, GH73, GH74, GH76, GH92, GH93, GH97, GT19, GT1, GT21, GT27, GT28, GT35, GT39, GT3, GT40, GT41, GT45, GT4, GT51, GT5, GT81, GT83, GT9, PL22, PL6, GT94, AA2, CBM67, CBM69, GH158, GH160, PL31, GT2_Glycos_transf_2, GT2_Glyco_tranf_2_2, GT2_Glyco_tranf_2_3, GT2_Glyco_tranf_2_4, GT2_Glyco_tranf_2_5, GT2_Glyco_trans_2_3, AA1_1, AA1_2, AA1_3, AA3_1, AA3_2, GH13_10, GH13_11, GH13_12, GH13_13, GH13_14, GH13_15, GH13_16, GH13_17, GH13_18, GH13_19, GH13_1, GH13_20, GH13_21, GH13_22, GH13_23, GH13_24, GH13_26, GH13_27, GH13_28, GH13_29, GH13_2, GH13_30, GH13_31, GH13_32, GH13_34, GH13_35, GH13_36, GH13_37, GH13_38, GH13_39, GH13_3, GH13_40, GH13_41, GH13_42, GH13_4, GH13_5, GH13_6, GH13_7, GH13_8, GH13_9, GH5_19, GH5_42, GH5_7, PL22_1, PL22_2, PL6_1, PL6_2, PL9_2, PL9_3, AA1, GH109, GT105, GH171, PL41* |
| bin. 17 | *CBM13, CBM50, CE16, CE1, CE4, CE7, CE9, cohesin, GH102, GH103, GH114, GH23, GH24, GH33, GH3, GH73, GH74, GT1, GT27, GT28, GT30, GT41, GT4, GT51, GT5, GT81, GT83, GT9, PL22, PL9, AA2, AA3, AA4, AA6, AA7, GT104, PL31, GT2_Glycos_transf_2, GT2_Glyco_tranf_2_2, GT2_Glyco_tranf_2_3, GT2_Glyco_tranf_2_5, AA3_1, AA3_2, AA3_3, AA3_4, GH5_35, PL22_1, GH166, GH109, CE19, GT112* |
| bin. 18 | *CBM11, CBM12, CBM16, CBM20, CBM22, CBM23, CBM32, CBM40, CBM48, CBM4, CBM50, CBM56, CBM6, CBM8, CBM9, CE11, CE14, CE1, CE3, CE4, CE6, CE7, CE9, cohesin, GH100, GH102, GH106, GH10, GH113, GH119, GH120, GH121, GH13,* |

| | |
|---|---|
| | *GH16, GH18, GH1, GH20, GH23, GH25, GH29, GH2, GH30, GH31, GH32, GH33, GH36, GH37, GH38, GH3, GH43, GH53, GH57, GH5, GH63, GH65, GH67, GH70, GH73, GH74, GH76, GH78, GH87, GH92, GH93, GH94, GH95, GH97, GH9, GT1, GT20, GT21, GT27, GT30, GT35, GT39, GT40, GT41, GT45, GT4, GT51, GT5, GT66, GT81, GT83, GT9, PL11, PL12, PL15, PL17, PL22, PL9, GH127, GH130, AA2, AA3, AA5, CBM66, CBM67, AA12, GH133, GH136, GH144, GH146, GH147, GH148, GH149, GH150, GH151, GH157, GH158, GH161, PL31, PL33, PL35, GT2_Glycos_transf_2, GT2_Glyco_tranf_2_2, GT2_Glyco_tranf_2_3, GT2_Glyco_tranf_2_4, GT2_Glyco_tranf_2_5, GT2_Glyco_trans_2_3, AA3_1, AA3_2, AA3_3, AA3_4, AA5_2, GH13_10, GH13_11, GH13_12, GH13_13, GH13_14, GH13_16, GH13_17, GH13_18, GH13_19, GH13_1, GH13_20, GH13_21, GH13_22, GH13_23, GH13_25, GH13_26, GH13_27, GH13_28, GH13_29, GH13_2, GH13_30, GH13_31, GH13_32, GH13_34, GH13_35, GH13_36, GH13_37, GH13_38, GH13_39, GH13_3, GH13_40, GH13_41, GH13_42, GH13_4, GH13_5, GH13_6, GH13_7, GH13_8, GH13_9, GH30_1, GH30_2, GH30_3, GH30_4, GH30_5, GH30_6, GH30_7, GH30_8, GH30_9, GH5_18, GH5_19, GH5_1, GH5_22, GH5_25, GH5_30, GH5_31, GH5_41, GH5_42, GH5_7, GH5_8, PL12_1, PL12_2, PL12_3, PL15_1, PL17_1, PL22_1, PL22_2, PL33_1, PL6_2, PL9_2, CBM85, CBM35, PL39, GH16_11, GH16_12, GH16_13, GH16_14, GH16_17, GH16_18, GH16_19, GH16_1, GH16_21, GH16_24, GH16_25, GH16_27, GH16_2, GH16_3, GH16_4, GH16_5, GH16_6, GH16_7, GH16_8, GH16_9, GH109, GH27, GT105, CBM88, CE19, GH171* |
| bin. 20 | *CBM16, CBM22, CBM32, CBM40, CBM4, CBM50, CBM9, CE11, CE12, CE14, CE1, CE2, CE3, CE4, CE7, CE9, GH105, GH120, GH16, GH18, GH20, GH23, GH24, GH29, GH2, GH33, GH35, GH39, GH3, GH42, GH5, GH73, GH74, GH76, GH79, GH92, GH93, GT11, GT12, GT19, GT21, GT22, GT26, GT27, GT28, GT30, GT32, GT39, GT41, GT45, GT4, GT51, GT5, GT76, GT81, GT83, GT9, PL10, PL22, GT94, AA2, AA4, AA7, CBM66, AA12, GH136, GH141, GH160, GH163, PL31, PL37, GT2_Glycos_transf_2, GT2_Glyco_tranf_2_2, GT2_Glyco_tranf_2_3, GT2_Glyco_tranf_2_4, GT2_Glyco_tranf_2_5, GT2_Glyco_trans_2_3, AA1_1, AA1_2, AA1_3, AA3_1, GH5_19, GH5_1, GH5_42, GH5_46, GH5_7, PL10_1, PL22_2, PL6_2, PL9_2, PL9_4, GH16_13, GH16_14, GH16_17, GH16_24, GH16_3, GH16_5, GH16_7, CE17, AA1, GH109, CBM88, CE19, GH171, PL41* |
| bin. 21 | *CBM32, CBM50, CE12, CE14, CE1, CE2, CE3, CE4, CE7, CE9, GH120, GH20, GH23, GH24, GH29, GH3, GH63, GH74, GH91, GH92, GT17, GT1, GT21, GT22, GT27, GT28, GT30, GT39, GT41, GT45, GT4, GT51, GT5, GT66, GT76, GT81, GT83, GT87, GT8, PL22, GT94, AA2, AA3, AA4, AA7, CBM66, CBM67, GH133, GH136, PL31, GT2_Glycos_transf_2, GT2_Glyco_tranf_2_2, GT2_Glyco_tranf_2_3, GT2_Glyco_tranf_2_4, GT2_Glyco_trans_2_3, AA1_1, AA1_2, AA1_3, AA3_1, AA3_2, PL9_2, AA1,* |

| | |
|---|---|
| | *GH109, GT105, CBM88, CE19, PL41* |
| bin. 22 | *CBM50, CE11, CE1, CE2, CE3, CE4, CE7, CE8, CE9, GH100, GH116, GH119, GH13, GH20, GH23, GH33, GH37, GH42, GH57, GH63, GH65, GH74, GH77, GH78, GH93, GH94, GH95, GT20, GT21, GT27, GT28, GT35, GT41, GT45, GT4, GT51, GT5, GT70, GT81, GT83, GT87, PL10, PL11, PL14, PL4, PL6, PL9, AA4, AA5, AA7, CBM66, GH133, GH142, GT2_Chitin_synth_2, GT2_Glycos_transf_2, GT2_Glyco_tranf_2_2, GT2_Glyco_tranf_2_3, GT2_Glyco_tranf_2_4, GT2_Glyco_trans_2_3, AA1_2, AA1_3, AA3_1, AA5_2, GH13_10, GH13_11, GH13_12, GH13_13, GH13_14, GH13_20, GH13_23, GH13_25, GH13_32, GH13_36, GH13_37, GH13_41, GH13_5, GH13_8, GH13_9, PL10_1, PL10_2, PL11_1, PL14_3, PL6_1, PL9_1, PL9_2, PL9_3, AA1, GH109, PL41* |
| bin. 23 | *CBM13, CBM32, CBM4, CBM50, CE11, CE1, CE3, CE4, CE7, CE9, GH103, GH114, GH17, GH23, GH33, GH3, GH42, GH5, GH73, GH74, GH93, GT19, GT21, GT26, GT28, GT30, GT41, GT51, GT81, GT9, PL22, AA3, AA4, AA6, AA7, GT104, GT2_Cellulose_synt, GT2_Glycos_transf_2, GT2_Glyco_tranf_2_3, GT2_Glyco_trans_2_3, AA1_2, AA1_3, AA3_1, AA3_2, AA3_3, AA3_4, GH5_12, GH5_19, GH5_1, GH5_22, GH5_24, GH5_25, GH5_26, GH5_27, GH5_28, GH5_29, GH5_2, GH5_37, GH5_39, GH5_40, GH5_43, GH5_46, GH5_47, GH5_48, GH5_4, GH5_51, GH5_54, GH5_5, GH5_7, GH5_8, PL22_1, GH166, AA1* |
| bin. 24 | *CE1, CE2, CE3, CE4, CE7, CE9, GH102, GH103, GH16, GH23, GH25, GH30, GH36, GH3, GH42, GH4, GH5, GT1, GT21, GT25, GT26, GT27, GT28, GT32, GT41, GT4, GT51, GT5, GT81, GT83, PL12, PL22, PL9, GT94, AA3, AA4, AA6, AA7, AA12, PL31, GT2_Glycos_transf_2, GT2_Glyco_tranf_2_2, GT2_Glyco_tranf_2_3, GT2_Glyco_trans_2_3, AA1_1, AA1_2, AA1_3, AA3_1, AA3_2, AA3_3, AA3_4, GH30_3, PL12_3, PL22_1, PL22_2, PL9_1, GH167, GH16_10, GH16_11, GH16_12, GH16_14, GH16_17, GH16_18, GH16_1, GH16_21, GH16_24, GH16_25, GH16_2, GH16_3, GH16_4, GH16_5, GH16_6, GH16_7, GH16_8, GH16_9, AA1, GH109, CE19, GH171* |
| bin. 28 | *CBM48, CBM50, CE12, CE1, CE3, CE4, CE7, GH102, GH103, GH13, GH23, GH30, GH3, GH73, GT19, GT21, GT27, GT28, GT30, GT35, GT39, GT40, GT41, GT45, GT4, GT51, GT5, GT81, GT83, GT9, PL22, AA3, AA4, AA6, AA7, GT104, GT2_Glycos_transf_2, GT2_Glyco_tranf_2_2, GT2_Glyco_tranf_2_3, GT2_Glyco_tranf_2_4, GT2_Glyco_tranf_2_5, GT2_Glyco_trans_2_3, AA3_1, AA3_2, AA3_3, AA3_4, GH13_10, GH13_11, GH13_12, GH13_13, GH13_14, GH13_16, GH13_17, GH13_19, GH13_1, GH13_20, GH13_21, GH13_23, GH13_28, GH13_29, GH13_2, GH13_30, GH13_31, GH13_32, GH13_35, GH13_36, GH13_37, GH13_38, GH13_39, GH13_3, GH13_40, GH13_41, GH13_42, GH13_4, GH13_5, GH13_6,* |

| | |
|---|---|
| | *GH13_8, GH13_9, GH30_1, GH30_2, GH30_3, GH30_4, GH30_5, GH30_6, GH30_7, GH30_8, PL6_1, PL6_2, CE19, GT112* |
| bin. 29 | *CBM20, CBM50, CE14, CE1, CE3, CE4, CE7, CE9, GH103, GH120, GH13, GH15, GH1, GH23, GH2, GH31, GH32, GH33, GH36, GH37, GH3, GH43, GH5, GH63, GH74, GH77, GH78, GH93, GH94, GT1, GT21, GT27, GT28, GT33, GT35, GT3, GT40, GT41, GT45, GT4, GT51, GT5, GT66, GT73, GT81, GT83, GT87, PL12, PL15, PL17, PL22, PL6, PL9, GT94, AA2, AA3, AA4, AA7, CBM66, CBM67, GH142, GH149, GH161, PL31, GT2_Glycos_transf_2, GT2_Glyco_tranf_2_2, GT2_Glyco_tranf_2_3, GT2_Glyco_trans_2_3, AA1_1, AA1_2, AA1_3, AA3_1, AA3_2, AA3_3, AA3_4, GH13_10, GH13_11, GH13_12, GH13_13, GH13_14, GH13_15, GH13_16, GH13_17, GH13_18, GH13_19, GH13_1, GH13_20, GH13_21, GH13_22, GH13_23, GH13_26, GH13_27, GH13_28, GH13_29, GH13_2, GH13_30, GH13_31, GH13_32, GH13_34, GH13_35, GH13_36, GH13_37, GH13_38, GH13_39, GH13_3, GH13_40, GH13_41, GH13_42, GH13_4, GH13_5, GH13_6, GH13_7, GH13_8, GH13_9, GH43_18, GH43_19, GH5_19, GH5_5, PL12_1, PL12_2, PL12_3, PL22_1, PL22_2, PL6_1, PL6_2, PL9_1, PL9_2, PL9_3, PL9_4, PL39, AA1, GH109, GH27, CE19, PL41* |
| bin. 30 | *CBM11, CBM13, CBM20, CBM40, CBM41, CBM48, CBM50, CBM9, CE14, CE16, CE1, CE3, CE4, CE7, CE8, CE9, cohesin, GH100, GH106, GH10, GH116, GH117, GH119, GH13, GH15, GH16, GH18, GH1, GH20, GH2, GH32, GH33, GH35, GH39, GH3, GH42, GH43, GH4, GH51, GH5, GH71, GH74, GH76, GH77, GH78, GH93, GH94, GH99, GT1, GT20, GT21, GT26, GT27, GT28, GT35, GT39, GT3, GT40, GT41, GT45, GT4, GT51, GT57, GT5, GT66, GT76, GT81, GT83, GT9, PL22, PL9, GH128, GH130, GT94, AA3, AA6, CBM66, CBM67, GH133, GH137, GH147, GH148, GH149, GH158, GH161, GH165, GT2_Glycos_transf_2, GT2_Glyco_tranf_2_2, GT2_Glyco_tranf_2_3, GT2_Glyco_tranf_2_4, GT2_Glyco_trans_2_3, AA3_1, AA3_2, GH13_10, GH13_11, GH13_12, GH13_13, GH13_14, GH13_15, GH13_16, GH13_17, GH13_18, GH13_19, GH13_1, GH13_20, GH13_21, GH13_22, GH13_23, GH13_24, GH13_25, GH13_26, GH13_27, GH13_28, GH13_29, GH13_2, GH13_30, GH13_31, GH13_32, GH13_34, GH13_35, GH13_36, GH13_37, GH13_38, GH13_39, GH13_3, GH13_40, GH13_41, GH13_42, GH13_4, GH13_5, GH13_6, GH13_7, GH13_8, GH13_9, GH43_18, GH43_23, GH43_32, GH5_19, GH5_30, GH5_41, GH5_7, PL22_1, PL9_2, GH16_10, GH16_11, GH16_12, GH16_13, GH16_14, GH16_17, GH16_1, GH16_21, GH16_24, GH16_2, GH16_3, GH16_4, GH16_5, GH16_6, GH16_7, GH16_8, GH16_9, GH109, CE19* |
| bin. 34 | *CBM16, CBM32, CBM48, CBM50, CBM9, CE11, CE12, CE14, CE1, CE3, CE4, CE7, CE8, CE9, cohesin, GH100, GH105, GH119, GH13, GH15, GH18, GH20, GH23, GH28, GH32, GH33, GH36, GH37, GH38, GH3, GH43, GH46, GH47, GH57, GH5, GH63, GH70, GH74, GH76, GH77, GH78, GH82, GH92, GH93, GH94, GH97, GH9, GT12, GT19, GT1, GT21, GT27, GT28, GT30,* |

| | |
|---|---|
| | *GT33, GT35, GT39, GT41, GT45, GT4, GT51, GT5, GT76, GT81, GT83, GT87, GT9, PL10, PL12, PL15, PL17, PL1, PL22, AA2, AA3, AA4, AA6, AA7, CBM66, AA12, CBM70, GH133, GH136, GH144, GH151, GH158, PL33, GT2_Chitin_synth_2, GT2_Glycos_transf_2, GT2_Glyco_tranf_2_2, GT2_Glyco_tranf_2_3, GT2_Glyco_tranf_2_4, GT2_Glyco_trans_2_3, AA3_1, AA3_2, AA3_4, GH13_10, GH13_11, GH13_12, GH13_13, GH13_14, GH13_15, GH13_16, GH13_17, GH13_18, GH13_19, GH13_1, GH13_20, GH13_21, GH13_22, GH13_23, GH13_25, GH13_26, GH13_27, GH13_28, GH13_29, GH13_2, GH13_30, GH13_31, GH13_32, GH13_34, GH13_35, GH13_36, GH13_37, GH13_38, GH13_39, GH13_3, GH13_40, GH13_41, GH13_42, GH13_4, GH13_5, GH13_6, GH13_7, GH13_8, GH13_9, GH43_10, GH43_11, GH43_12, GH43_13, GH43_14, GH43_15, GH43_16, GH43_17, GH43_18, GH43_19, GH43_1, GH43_22, GH43_24, GH43_26, GH43_28, GH43_29, GH43_2, GH43_30, GH43_31, GH43_32, GH43_33, GH43_34, GH43_36, GH43_37, GH43_3, GH43_4, GH43_5, GH43_8, GH43_9, GH5_10, GH5_19, GH5_22, GH5_30, GH5_31, GH5_41, GH5_42, GH5_46, GH5_7, PL10_1, PL10_2, PL10_3, PL11_2, PL12_1, PL12_2, PL12_3, PL15_2, PL17_1, PL22_1, PL22_2, PL33_1, PL9_2, PL9_4, PL39, CE17, GH109, CE19, GH171* |
| bin. 35 | *CBM16, CBM32, CBM48, CBM50, CBM56, CBM9, CE11, CE12, CE14, CE15, CE1, CE3, CE4, CE6, CE7, CE9, GH105, GH106, GH10, GH110, GH113, GH116, GH120, GH123, GH13, GH16, GH18, GH1, GH20, GH23, GH28, GH29, GH2, GH31, GH33, GH36, GH38, GH39, GH3, GH42, GH44, GH50, GH51, GH55, GH57, GH5, GH65, GH67, GH70, GH74, GH77, GH78, GH88, GH93, GH94, GH95, GH9, GT19, GT1, GT20, GT21, GT27, GT28, GT35, GT39, GT41, GT45, GT4, GT51, GT5, GT81, GT83, GT84, GT9, PL11, PL12, PL15, PL17, PL22, PL9, GH127, GH130, AA3, AA7, CBM66, CBM67, CBM70, GH136, GH140, GH141, GH143, GH146, GH151, GH158, GH160, GH164, PL31, PL33, PL35, GT2_Glycos_transf_2, GT2_Glyco_tranf_2_2, GT2_Glyco_tranf_2_3, GT2_Glyco_trans_2_3, AA1_1, AA1_2, AA1_3, AA3_1, AA3_2, AA3_3, AA3_4, GH13_10, GH13_11, GH13_12, GH13_13, GH13_14, GH13_15, GH13_16, GH13_17, GH13_18, GH13_19, GH13_1, GH13_20, GH13_21, GH13_22, GH13_23, GH13_24, GH13_26, GH13_27, GH13_28, GH13_29, GH13_2, GH13_30, GH13_31, GH13_32, GH13_34, GH13_35, GH13_36, GH13_37, GH13_38, GH13_39, GH13_3, GH13_40, GH13_41, GH13_42, GH13_4, GH13_5, GH13_6, GH13_7, GH13_8, GH13_9, GH5_10, GH5_13, GH5_14, GH5_17, GH5_19, GH5_1, GH5_22, GH5_25, GH5_26, GH5_29, GH5_2, GH5_36, GH5_37, GH5_38, GH5_39, GH5_44, GH5_45, GH5_46, GH5_47, GH5_48, GH5_4, GH5_50, GH5_52, GH5_54, GH5_5, GH5_7, GH5_8, GH5_9, PL11_1, PL12_3, PL22_1, PL22_2, PL33_1, PL33_2, PL9_1, PL9_2, PL9_3, GH167, PL38, PL39, GH16_10, GH16_11, GH16_12, GH16_13, GH16_14, GH16_17, GH16_18, GH16_19, GH16_1, GH16_20, GH16_21, GH16_23, GH16_24, GH16_25, GH16_27, GH16_2, GH16_3, GH16_4, GH16_5, GH16_6, GH16_7, GH16_8, GH16_9, CE17,* |

| | |
|---|---|
| | *AA1, GH109, CE19, GH171, PL41* |
| bin. 36 | *CBM11, CBM16, CBM23, CBM32, CBM47, CBM48, CBM50, CBM57, CBM61, CBM6, CBM8, CBM9, CE11, CE12, CE14, CE1, CE2, CE3, CE4, CE7, CE9, GH100, GH106, GH119, GH13, GH23, GH31, GH35, GH39, GH3, GH42, GH51, GH57, GH5, GH76, GH78, GH79, GH94, GT19, GT20, GT21, GT27, GT28, GT30, GT35, GT39, GT40, GT41, GT45, GT4, GT51, GT5, GT81, GT83, GT84, GT9, PL22, AA2, AA3, AA4, AA7, GH133, GH144, GT2_Glycos_transf_2, GT2_Glyco_tranf_2_2, GT2_Glyco_tranf_2_3, GT2_Glyco_tranf_2_4, GT2_Glyco_trans_2_3, AA1_2, AA1_3, AA3_1, AA3_2, AA3_3, AA3_4, GH13_25, GH13_30, GH13_32, GH13_3, PL22_1, PL22_2, CBM35, GH167, AA1, CBM30, GH109, GT105, CE19, GH171, GT112* |
| bin. 37 | *CBM50, CE11, CE14, CE1, CE3, CE7, CE9, GH102, GH103, GH23, GH3, GH73, GH8, GT19, GT21, GT27, GT28, GT30, GT41, GT42, GT4, GT51, GT73, GT81, GT83, GT8, PL22, PL6, PL9, AA3, AA4, AA5, AA6, AA7, GT104, PL31, GT2_Cellulose_synt, GT2_Glycos_transf_2, GT2_Glyco_tranf_2_2, GT2_Glyco_tranf_2_3, GT2_Glyco_tranf_2_5, GT2_Glyco_trans_2_3, AA1_1, AA1_2, AA1_3, AA3_1, AA3_2, PL22_1, CE17, AA1* |
| bin. 39 | *CBM32, CBM37, CBM40, CBM50, CBM56, CBM9, CE11, CE14, CE2, CE3, CE4, CE7, CE9, cohesin, GH18, GH20, GH23, GH28, GH2, GH33, GH3, GH55, GH74, GH92, GH93, GT13, GT19, GT1, GT21, GT22, GT27, GT28, GT30, GT33, GT39, GT40, GT41, GT45, GT4, GT51, GT57, GT58, GT5, GT75, GT77, GT81, GT83, GT87, GT9, PL12, PL17, PL22, PL6, GT94, AA3, AA4, AA6, AA7, CBM67, PL42, GH150, GH158, PL29, PL31, GT2_Chitin_synth_2, GT2_Glycos_transf_2, GT2_Glyco_tranf_2_2, GT2_Glyco_tranf_2_3, GT2_Glyco_tranf_2_4, GT2_Glyco_tranf_2_5, GT2_Glyco_trans_2_3, AA1_1, AA1_2, AA1_3, AA3_1, AA5_2, PL12_1, PL12_3, PL22_1, PL9_2, CBM35, AA1, GH109, GT105, CBM88, GH171* |
| bin. 40 | *CBM48, CBM50, CE1, CE3, CE4, CE7, CE8, CE9, GH100, GH116, GH117, GH119, GH13, GH15, GH23, GH26, GH32, GH33, GH37, GH39, GH3, GH43, GH57, GH5, GH63, GH74, GH77, GH78, GH93, GH94, GT12, GT20, GT21, GT27, GT35, GT39, GT40, GT41, GT45, GT4, GT51, GT5, GT81, GT83, GT84, GT9, PL12, PL17, PL1, PL22, PL4, PL6, PL9, GT94, AA3, AA4, AA6, AA7, CBM66, GH133, GH136, GH142, GH144, GH159, GT2_Glycos_transf_2, GT2_Glyco_tranf_2_2, GT2_Glyco_tranf_2_3, GT2_Glyco_tranf_2_4, GT2_Glyco_tranf_2_5, AA1_1, AA1_2, AA1_3, AA3_1, GH13_10, GH13_11, GH13_12, GH13_13, GH13_14, GH13_16, GH13_17, GH13_19, GH13_1, GH13_20, GH13_21, GH13_23, GH13_25, GH13_26, GH13_27, GH13_28, GH13_29, GH13_2, GH13_30, GH13_31, GH13_32, GH13_35, GH13_36, GH13_37, GH13_38, GH13_39, GH13_3, GH13_40, GH13_41, GH13_42, GH13_4, GH13_5, GH13_6, GH13_7, GH13_8, GH13_9, GH43_18, GH43_9, PL12_1, PL12_2, PL12_3, PL1_2, PL22_1, PL22_2, PL9_1, PL9_2, PL9_3, AA1, GH109, PL41* |

| | |
|---|---|
| bin. 41 | *CBM34, CBM48, CBM50, CE11, CE1, CE4, CE7, CE8, CE9, GH102, GH103, GH105, GH13, GH1, GH23, GH24, GH28, GH2, GH31, GH32, GH36, GH37, GH3, GH43, GH4, GH5, GH65, GH73, GH77, GH78, GH94, GT19, GT1, GT20, GT21, GT26, GT27, GT28, GT30, GT35, GT39, GT41, GT45, GT4, GT51, GT56, GT5, GT81, GT83, GH127, GH130, AA2, AA3, AA4, AA6, AA7, GH142, GH146, GH153, GH156, GH158, GH159, GT2_Chitin_synth_2, GT2_Glycos_transf_2, GT2_Glyco_tranf_2_2, GT2_Glyco_tranf_2_3, GT2_Glyco_trans_2_3, AA1_1, AA1_2, AA1_3, AA3_1, AA3_2, GH13_10, GH13_11, GH13_12, GH13_13, GH13_14, GH13_15, GH13_16, GH13_17, GH13_18, GH13_19, GH13_1, GH13_20, GH13_21, GH13_22, GH13_23, GH13_26, GH13_27, GH13_28, GH13_29, GH13_2, GH13_30, GH13_31, GH13_32, GH13_33, GH13_34, GH13_35, GH13_36, GH13_37, GH13_38, GH13_39, GH13_3, GH13_40, GH13_41, GH13_42, GH13_4, GH13_5, GH13_6, GH13_7, GH13_8, GH13_9, GH43_10, GH43_11, GH43_12, GH43_17, GH43_18, GH43_19, GH43_22, GH43_23, GH43_26, GH43_27, GH43_28, GH43_29, GH43_30, GH43_31, GH43_32, GH43_33, GH43_34, GH43_3, GH43_4, GH43_5, GH43_6, GH43_8, GH43_9, GH5_22, GH5_25, GH5_28, GH5_36, GH5_37, GH5_46, GH5_4, GH5_54, GH5_8, AA1, GH109* |
| bin. 44 | *CBM50, CE11, CE16, CE1, CE2, CE3, CE4, CE7, CE9, cohesin, GH102, GH17, GH23, GH5, GH74, GT19, GT1, GT21, GT27, GT28, GT30, GT39, GT40, GT41, GT45, GT4, GT51, GT5, GT81, GT83, GT9, PL1, PL22, PL6, PL9, AA3, AA4, AA6, AA7, AA12, PL31, GT2_Glycos_transf_2, GT2_Glyco_tranf_2_2, GT2_Glyco_tranf_2_3, GT2_Glyco_trans_2_3, AA3_1, AA3_2, AA3_3, GH5_42, PL11_2, PL22_1, PL6_1, PL6_2, PL9_2, CE17, GH171, PL41* |
| bin. 48 | *CBM16, CBM20, CBM34, CBM48, CBM50, CE14, CE1, CE4, CE7, CE9, GH100, GH105, GH116, GH119, GH13, GH15, GH18, GH1, GH23, GH30, GH33, GH35, GH37, GH38, GH39, GH3, GH42, GH4, GH51, GH57, GH5, GH63, GH71, GH74, GH78, GH79, GH93, GH94, GH99, GT12, GT1, GT21, GT26, GT27, GT28, GT33, GT35, GT39, GT3, GT40, GT41, GT45, GT4, GT51, GT5, GT66, GT81, GT83, GT9, PL11, PL22, PL9, GH128, GT94, AA3, AA4, AA5, AA6, AA7, CBM66, CBM67, GH133, GH136, GH142, GH144, GH150, GH151, GT2_Glycos_transf_2, GT2_Glyco_tranf_2_2, GT2_Glyco_tranf_2_3, GT2_Glyco_tranf_2_4, GT2_Glyco_trans_2_3, AA3_1, AA3_2, AA5_2, GH13_10, GH13_11, GH13_12, GH13_13, GH13_14, GH13_15, GH13_16, GH13_17, GH13_18, GH13_19, GH13_1, GH13_20, GH13_21, GH13_22, GH13_23, GH13_24, GH13_25, GH13_26, GH13_27, GH13_28, GH13_29, GH13_2, GH13_30, GH13_31, GH13_32, GH13_34, GH13_35, GH13_36, GH13_37, GH13_38, GH13_39, GH13_3, GH13_40, GH13_41, GH13_42, GH13_4, GH13_5, GH13_6, GH13_7, GH13_8, GH13_9, GH5_19, GH5_28, GH5_29, GH5_47, GH5_5, PL11_1, PL22_1, PL9_1, PL9_2, GH109, GT105, CE19* |
| bin. 49 | *CBM11, CBM23, CBM48, CBM50, CE1, CE7, CE9, GH100, GH102, GH23, GH35, GH3, GH42, GH57, GH74, GH77, GH78,* |

| | |
|---|---|
| | GH94, GT19, GT1, GT21, GT26, GT27, GT28, GT35, GT39, GT41, GT4, GT51, GT5, GT81, GT83, GT84, GT9, PL22, AA2, AA3, AA4, AA5, AA7, GH133, GH142, GH144, GT2_Chitin_synth_2, GT2_Glycos_transf_2, GT2_Glyco_tranf_2_2, GT2_Glyco_tranf_2_3, GT2_Glyco_tranf_2_4, GT2_Glyco_trans_2_3, AA3_1, GH13_25, PL22_1, GH167, GH109, GH171 |
| bin. 52 | CBM48, CBM50, CBM9, CE11, CE1, CE2, CE3, CE4, CE7, CE9, GH102, GH103, GH10, GH117, GH121, GH13, GH15, GH23, GH32, GH33, GH3, GH55, GH57, GH73, GH74, GH77, GH78, GH93, GT19, GT1, GT20, GT25, GT26, GT27, GT28, GT30, GT35, GT41, GT4, GT51, GT5, GT81, GT9, PL12, PL22, GH130, AA3, AA4, AA5, AA7, CBM66, GH141, GT102, GT104, GH159, GH165, GT2_Glycos_transf_2, GT2_Glyco_tranf_2_3, GT2_Glyco_trans_2_3, AA1_1, AA1_2, AA1_3, AA3_1, AA3_2, AA3_3, AA3_4, AA5_1, AA5_2, GH13_10, GH13_11, GH13_12, GH13_13, GH13_14, GH13_15, GH13_16, GH13_17, GH13_18, GH13_19, GH13_1, GH13_20, GH13_21, GH13_22, GH13_23, GH13_26, GH13_27, GH13_28, GH13_29, GH13_2, GH13_30, GH13_31, GH13_32, GH13_34, GH13_35, GH13_36, GH13_37, GH13_38, GH13_39, GH13_3, GH13_40, GH13_41, GH13_42, GH13_4, GH13_5, GH13_6, GH13_7, GH13_8, GH13_9, GH43_30, PL12_3, PL22_1, PL22_2, PL6_1, AA1, GH109, GH171, PL41 |
| bin. 55 | CBM48, CBM50, CE11, CE1, CE3, CE4, CE7, CE9, GH102, GH103, GH108, GH113, GH117, GH13, GH1, GH20, GH23, GH25, GH2, GH37, GH3, GH43, GH4, GH55, GH5, GH63, GH78, GT14, GT19, GT1, GT21, GT25, GT26, GT28, GT30, GT32, GT35, GT41, GT4, GT51, GT5, GT81, GT92, PL22, AA3, AA4, AA7, CBM67, GT99, GT107, PL31, GT2_Glycos_transf_2, GT2_Glyco_tranf_2_3, GT2_Glyco_tranf_2_4, GT2_Glyco_trans_2_3, AA3_1, AA3_2, AA3_3, AA3_4, GH13_10, GH13_11, GH13_12, GH13_13, GH13_14, GH13_16, GH13_17, GH13_18, GH13_19, GH13_1, GH13_20, GH13_21, GH13_22, GH13_23, GH13_26, GH13_27, GH13_28, GH13_29, GH13_2, GH13_30, GH13_31, GH13_32, GH13_34, GH13_35, GH13_36, GH13_37, GH13_38, GH13_39, GH13_3, GH13_40, GH13_41, GH13_42, GH13_4, GH13_5, GH13_6, GH13_7, GH13_8, GH13_9, GH43_10, GH43_11, GH43_12, GH43_13, GH43_14, GH43_15, GH43_16, GH43_18, GH43_19, GH43_23, GH43_24, GH43_26, GH43_28, GH43_29, GH43_2, GH43_30, GH43_31, GH43_32, GH43_33, GH43_34, GH43_36, GH43_37, GH43_3, GH43_4, GH43_5, GH43_8, GH43_9, GH5_25, GH5_36, GH5_37, GH5_38, GH5_39, GH5_46, GH5_48, GH5_52, PL12_3, PL22_1, PL6_1, GH109 |
| bin. 56 | CBM48, CBM50, CE11, CE12, CE1, CE3, CE4, CE7, CE9, GH102, GH103, GH13, GH15, GH16, GH17, GH23, GH24, GH30, GH33, GH63, GH73, GH74, GH77, GH81, GH84, GH93, GT19, GT25, GT27, GT28, GT30, GT35, GT39, GT41, GT4, GT51, GT5, GT81, GT83, GT9, PL22, AA2, AA3, AA4, AA5, AA6, AA7, GH149, GH161, GT2_Glycos_transf_2, GT2_Glyco_tranf_2_2, GT2_Glyco_tranf_2_3, GT2_Glyco_trans_2_3, AA3_1, AA3_2, AA3_3, AA3_4, AA5_2, GH13_10, GH13_11, GH13_12, GH13_13, |

| | |
|---|---|
| | *GH13_14, GH13_16, GH13_17, GH13_19, GH13_1, GH13_20, GH13_21, GH13_23, GH13_26, GH13_28, GH13_29, GH13_2, GH13_30, GH13_31, GH13_32, GH13_36, GH13_37, GH13_38, GH13_39, GH13_3, GH13_40, GH13_41, GH13_42, GH13_4, GH13_5, GH13_6, GH13_8, GH13_9, GH30_1, GH30_2, GH30_3, GH30_6, GH30_7, GH30_8, GH30_9, GH16_11, GH16_12, GH16_13, GH16_14, GH16_17, GH16_19, GH16_1, GH16_21, GH16_24, GH16_25, GH16_2, GH16_3, GH16_4, GH16_5, GH16_6, GH16_7, GH16_8, GH16_9, GH109, CE19* |
